# Activation of immune signals during organ transplantation

**DOI:** 10.1038/s41392-023-01377-9

**Published:** 2023-03-11

**Authors:** Qingwen Li, Peixiang Lan

**Affiliations:** 1 Institute of Organ Transplantation, Tongji Hospital, Tongji Medical College, Huazhong University of Science and Technology, Wuhan, 430030 China; 2 Key Laboratory of Organ Transplantation, Ministry of Education; NHC Key Laboratory of Organ Transplantation; Key Laboratory of Organ Transplantation, Chinese Academy of Medical Sciences, Wuhan, China

**Keywords:** Transplant immunology, Molecular medicine

## Abstract

The activation of host’s innate and adaptive immune systems can lead to acute and chronic graft rejection, which seriously impacts graft survival. Thus, it is particularly significant to clarify the immune signals, which are critical to the initiation and maintenance of rejection generated after transplantation. The initiation of response to graft is dependent on sensing of danger and stranger molecules. The ischemia and reperfusion of grafts lead to cell stress or death, followed by releasing a variety of damage-associated molecular patterns (DAMPs), which are recognized by pattern recognition receptors (PRRs) of host immune cells to activate intracellular immune signals and induce sterile inflammation. In addition to DAMPs, the graft exposed to ‘non-self’ antigens (stranger molecules) are recognized by the host immune system, stimulating a more intense immune response and further aggravating the graft damage. The polymorphism of MHC genes between different individuals is the key for host or donor immune cells to identify heterologous ‘non-self’ components in allogeneic and xenogeneic organ transplantation. The recognition of ‘non-self’ antigen by immune cells mediates the activation of immune signals between donor and host, resulting in adaptive memory immunity and innate trained immunity to the graft, which poses a challenge to the long-term survival of the graft. This review focuses on innate and adaptive immune cells receptor recognition of damage-associated molecular patterns, alloantigens and xenoantigens, which is described as danger model and stranger model. In this review, we also discuss the innate trained immunity in organ transplantation.

## Introduction

Transplantation rejection has always been the most critical problem affecting the long-term survival of allografts, which involves many biological processes. During transplantation, the graft experiences hypoxia/ischemia during preservation, leading to dysmetabolism and stress response. On the one hand, it induces the production of mediators related to inflammation and expands the inflammatory damage of cells. On the other hand, it mediates cells death. Damage-associated molecular patterns (DAMPs) from cells are exposed on the cell surface or released extracellular due to cellular stress or death. Graft reperfusion allows exposed DAMPs to be recognized by pattern recognition receptors (PRRs) of the host circulating immune cells. The interaction between DAMPs and PRRs activates PRRs, transmits activation signals intracellular, and stimulates immune cells to secrete pro-inflammatory cytokines and chemokines, resulting in aggravated graft injury. In addition to inducing innate immune response, the DAMPs produced by ischemia/reperfusion can be involved in the initiation of adaptive immunity as signal 0 via Dendritic cells (DCs).^1^ However, it is important to note that compared to allogeneic transplantation, DAMPs from syngeneic graft induce DCs production and promote T lymphocyte cloning and proliferation, but they are not enough to induce DCs to produce IL-12 and cannot drive T cells to differentiate into lymphocytes capable of producing IFN-γ.^2,3^ Therefore, it’s not sufficient to activate adaptive immune signals.

The host immune system distinguishes self and ‘non-self’ components, tolerates its healthy cells, and eliminates cells with ‘non-self’ antigens, which inevitably lead to graft rejection. To recognition of ‘non-self’ antigens, the host immune system requires the participation of receptors of immune cells. TCR of T and BCR of B lymphocytes can recognize allogeneic ‘non-self’ MHC antigens, leading to the activation of T and B lymphocyte-mediated rejection. However, the mere involvement of adaptive immune cells in recognizing ‘non-self’ MHC molecules cannot explain the fact that the depletion of lymphocytes still produces immune rejection of ‘non-self’ antigens.^3^ Multiple evidences reveal that receptors expressed in innate immune NK and myeloid cells can also recognize allogeneic antigens. These receptors belong to a family of immunoglobulin-like receptors (ILRs), which recognize MHC molecules including classical and non-classical MHC and are involved in the regulation of transplant tolerance and immune response. Based on the release of DAMPs induced by metabolic reprogramming and stress responses during transplantation, as well as the exposure of allogenic molecules to the host, this review mainly discusses the role of DAMPs, allogenic and xenogeneic ‘non-self’ component in immune recognition and induction of positive immune signals during transplantation.

## History of organ transplantation research

**Fig. 1:**
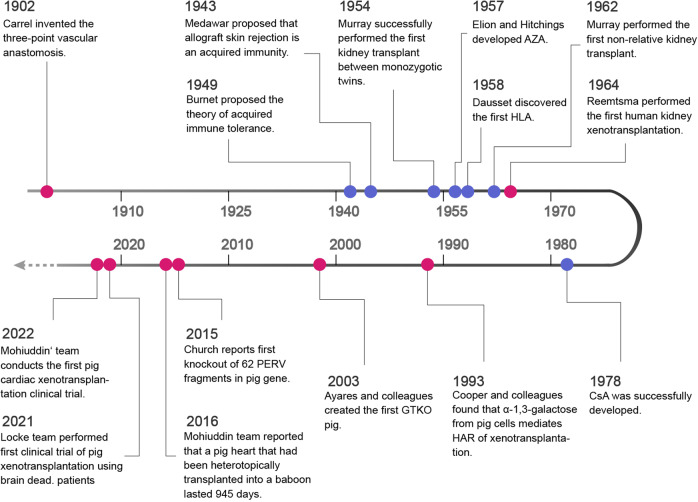
Historical timeline of organ transplantation. The blue dots are historical nodes related to transplantation immunity

The history of organ transplantation is inevitably associated with the development of immunology. Advancements in our understanding of immune rejection have also driven clinical application of organ transplantation. Organ transplantation, as a powerful treatment for end-stage diseases, has gone through a long and tortuous history (Fig. 1). As early as 1869, doctors attempted to transplant organ or tissues to patients. Dr. Reverdin, from France, grafted the skin of the patient’s raw granulation tissue to accelerate the healing of the wound. In 1883, The Swiss surgeon Theodor Kocher discovered that patients undergoing total thyroidectomy developed symptoms of hypothyroidism and childhood cretinism as defined by modern medicine. To address this complication, Kocher transplanted thyroid tissue into the patient. This is the first time to treat complex medical diseases by replacing organs. Although organ transplantation has its own original form, there are still many obstacles to be overcome.

The three cornerstones of organ transplantation are vascular anastomosis, short-term hypothermic organ preservation and inhibition of immune rejection. In the past, it was believed that it was impossible to suture between arteries and veins. It was not until 1902 that the French doctor Alexis Carrel invented the three-point vascular anastomosis method, which finally overcame the problem of vascular suture and laid a solid foundation for organ transplantation. Subsequently, different attempts were made in various organ transplantation operations. In 1933, the Ukrainian surgeon Voronoy performed the first human kidney transplantation in the Soviet Union. However, due to limited knowledge about immune rejection in humans, the kidneys used at that time were obtained 6 hours after the death of the donor, and the donor and recipient blood types were not matched. Thus, although the recipient survived for 2 days, the transplanted kidney did not produce urine. The operation ended in failure.

A turning point in the field of transplantation came in the 1930s. During this period, Medawar and Gibson collaborated on the study of skin grafting for burn patients in England. They performed a skin transplant on a woman with extensive burns, using both her own skin and her brother’s skin onto the burn site at the same time. Observing the effect of transplantation treatment, they found that the patient showed significant rejection of the ‘allogeneic’ skin graft. A large number of inflammatory leukocytes and lymphocytes infiltrated the rejected skin tissue. The intensity of rejection is proportional to the number of these inflammatory cells. And it shows obvious ‘memory’ phenomenon, with typical characteristics of immune response. However, the transplanted ‘autologous’ skin always grew well and did not appear this situation. In 1943, they published this research result and clearly pointed out that ‘the mechanism of allograft skin rejection is an active acquired immune response’. This is a landmark contribution in the field of transplant immunology. In 1945, Owen, a geneticist at the University of Wisconsin, reported that there were two types of red blood cells in the blood of dizygotic twin calves. Their own set of red cells and another set of red cells from their twin siblings. Red blood cells with different genetic backgrounds cannot coexist in the same animal. The presence of red blood cell mosaicism between calves of dizygotic twins suggests that the calves of these twins have developed immune tolerance to the red blood cells of the other calf of their sibling. The discovery intrigued Australian immunologist Burne, who was aware of the fact that twin cows undergo embryonic vascular fusion during the embryonic period and undergo blood exchange. In 1949, he proposed the hypothesis that the embryonic immune system was learning to recognize ‘self’ components, so that any antigen encountered at this time would be tolerated as ‘self’ component. Medawar did not observe rejection in the experiment of skin transplantation between heterozygotic twin calves, which he thought could be explained by Burnet ‘s theory. They then designed an experiment to artificially induce embryonic immune tolerance, which proved Burnet’s hypothesis that the immune system of embryonic animals develops immune tolerance in response to antigen stimulation. They call it ‘Acquired Immunologic Tolerance’.

Then, in 1954, American surgeon Murray successfully performed kidney transplantation between monozygotic twins and achieved long-term survival. This greatly encouraged the enthusiasm of surgeons for transplantation and officially opened the chapter of organ transplantation in modern medicine. In 1959, Murray and Hamburger performed kidney transplantation for dizygotic twins respectively. After the operation, the recipient received whole body radiation to control the immune rejection, and the patient achieved long-term survival. Despite the success of organ transplantation between twins, severe rejection still occurs in non-twin organ transplantation. The immune mechanism of graft rejection is complex. One of the most important reasons is that the donor organs carry different major histocompatibility antigens (MHC). Human MHC is called human leukocyte antigen (HLA). In 1958, French immunologist Jean Dausset discovered the human leucocyte antigen system by detecting 50 serum samples. Unmatched HLAs can be recognized by the immune system of the recipient and mediate hyperacute, accelerated, acute and chronic rejection. However, hyperacute and accelerated rejection rarely occur in donors and recipients with successful HLA matching after renal transplantation. Combined with the use of immunosuppressants, the survival rate of transplant patients can be significantly increased. Therefore, in 2001, the United States federal legislation included HLA matching in the technical standard of organ transplantation.

The research and development of immunosuppressants played a crucial role in the rapid development of organ transplantation in the following decades. In 1957, Elion and Hitchings exploited Azathioprine (AZA), a drug used to treat leukemia. It has purine antagonism. Through purine antagonism, it can inhibit the synthesis of DNA, RNA and protein, thus inhibiting the proliferation of lymphocytes. Researchers then applied AZA to allogeneic organ transplantation and found that AZA could reduce renal allograft rejection. In 1962, Murray performed the first successful cadaveric kidney transplant with the addition of AZA as an immunosuppressive drug. Later, from 1963 to 1967, the first lung, heart, liver and combined pancreas and renal transplantation was performed. Starzl and Goodwin found that AZA combined with corticosteroids, especially prednisone, had a synergistic effect. As a result, AZA combined with cortisol hormones became a routine standard regimen for immunosuppression in renal transplantation. Although AZA has a certain immunosuppressive effect, it has strong side effects such as bone marrow suppression. The 1-year survival rate of AZA combined with prednisone in renal transplantation is only about 50%, and it is not enough to achieve liver and other organ transplantation. Scientists began to search for other immunosuppressive drugs with good efficacy, high specificity and less toxic side effects. In 1969, the Swiss company Sandoz isolated a metabolite named Cyclosporine A (CsA) from soil samples of Tolypocladium inflatum. CsA is a calcineurin immunosuppressant (CNI), which can specifically inhibit the proliferation and reaction of lymphocytes, especially T lymphocytes. Its immunosuppressive effect was first discovered in 1972. Since then, further research and development of CsA has been carried out, including animal experiments and clinical trials. CsA was successfully developed and approved for clinical application in 1983. The therapeutic effect on immune rejection of allogeneic organ transplantation was rapidly improved. Since then, CsA combined with glucocorticoid has been used in kidney, liver, heart, lung, pancreas, bone marrow and other organs transplantation, and achieved satisfactory results.

Following CsA, in 1984, Tacronimus, also known as FK506, was isolated, with similar mechanisms of action to CsA but stronger T lymphocyte inhibitory effects and lower toxicity to the liver and kidneys. In 1989, FK506 was first used in organ transplantation and achieved remarkable clinical curative effect. It was subsequently approved for liver and kidney transplantation in 1994 and 1997, respectively. After FK506, a new immunosuppressive drug with similar structure to FK506 was found-Sirolimus, also known as Rapamycin (RAPA). But its mechanism of action was different from FK506. RAPA works on the late stage of T cell activation, inhibits the activation of T cells by blocking IL-2, blocks the process of T lymphocytes and other cells from G1 phase to S phase to exerts immunosuppressive effects. In 1999, RAPA was approved by the FDA for use in kidney transplants. In 1995, FDA approved the use of mycophenolatemofetil (MMF), which is used as an adjuvant medication of CsA to prevent acute rejection of renal transplantation. Mycophenolic acid (MPA), the active ingredient of MMF, is an inhibitor of Inosine-5’-monophosphate dehydrogenase (IMPDH) and guanylate synthase. IMPDH is the rate-limiting enzyme in the classical synthesis pathway of guanine. Lymphocytes cannot synthesize purine nucleotides through salvage pathways, but can only rely on classical synthesis pathways. Therefore, it can inhibit DNA synthesis and selectively inhibit lymphocyte proliferation. These immunosuppressants are usually used with glucocorticoids as an immune maintenance regimen after organ transplantation, showing excellent efficacy.

The application of immune anti-rejection drugs has greatly reduced the occurrence of rejection after transplantation, and has also made a qualitative leap in the quantity and quality of organ transplantation around the world. However, the increasing demand for organ transplantation and the shortage of donors has become a major concerned issue in the field of organ transplantation. Scientists are exploring alternative methods to obtain donor organs, including tissue engineering and heterogeneous animals. At present, the technical progress of tissue engineering is still limited to small animal experiments, making it difficult to achieve in the short term. Therefore, xenotransplantation is the most promising means to solve the problem of donor shortage.

From 1905 to 1993, surgeons successively performed renal, cardiac and liver xenotransplantation. The donors were rabbit, chimpanzee, baboon, goat, sheep and pig. but the curative effect was not ideal. The patient died soon after transplantation due to immune rejection and complications including infection. In 1964, the first successful kidney xenotransplantation was performed in the United States. Dr. Reemtsma implanted a baboon kidney into a patient, and the patient survived for 9 months after using immunosuppression. The success of this operation sparked scientists’ enthusiasm for xenotransplantation. Since then, heart and liver xenotransplantation have been performed. In the same year, Hardy performed the first human xenotransplantation of a heart from a chimpanzee. But the patient died two hours later. In 1966, Starzl performed the first liver xenotransplantation of chimpanzee liver into human, and the patient survived less than 1 day. In 1984, American doctor Bailey transplanted the heart of a female baboon into a baby with congenital heart malformation. The baby died of rejection 21 days after surgery. In 1992, Starzl completed the world ‘s first liver xenotransplantation of baboon liver to human. Unfortunately, the patient eventually died of infection after 72 days. Direct clinical application of xenogeneic organs was prohibited due to successive failures of xenotransplantation. Xenotransplantation started a long silent period.

The advent of CRISPR/Cas9 gene editing technology has brought xenotransplantation back into the sight of scientists. By evaluating the availability, reproductive capacity, anatomy, physiology, organ size and other elements between pigs and primates, pigs were finally determined to be the most ideal source of organs. Pig organs have many unknown epitopes, and their coagulation system is different from that of humans, plus some unpredictable biosecurity risks. It brings great challenges to xenotransplantation. Scientists try to knock out the genes that cause rejection to the humans, transfer genes that regulate immune and coagulation functions, and exclude pathogens from pigs as much as possible. The goal is to produce safe and effective genetically engineered animals.

In 1993, David Cooper and his colleagues found that the human immune system could initiate immune rejection within minutes after pig organ transplantation. The reason for this is the presence sof an antibody against α-1,3-galactose on the surface of pig cells in human. Hyperacute rejection can be triggered within a few hours after antigen-antibody combination, which is the initiating factor for rejection. In 2003, Revivicor founder Ayares and his colleagues created the first knockout-gal cloned transgenic pig (GTKO pig), which greatly advanced the field of xenotransplantation. Several research teams have reported multiplex gene edited pig organ transplantation experiments. In 2016, Mohiuddin’s research group reported success in xenotransplantation by knocking out α-1,3-galactose (GTKO) and transferring human thrombomodulin (hTBM) and complement regulatory protein (hCD46), and using anti-CD40 antibody treatment. The pig heart transplanted into the baboon was maintained for 2 and a half years.^4^ In 2017, Cooper’s team announced that a pig kidney engineered with six genes, combined with immunosuppressive therapy, had managed to keep a baboon alive for more than eight months. In 2015, George Church and Luhan Yang firstly reported the successful knockout of 62 PERV fragments in pig genes by using CRISPR/Cas9 gene editing technology.^5^ It reduces the infection rate of cultured human cells to one thousandth of the original. In 2020, the expert team of China Xijing Hospital made history by conducting the world’s first transplantation of organs from genetically modified pigs, including a liver, heart, and kidney, to three rhesus monkeys. This is the world ‘s longest surviving recipient of auxiliary liver xenotransplantation. In 2021, researchers at the University of Alabama at Birmingham (UAB) School of Medicine orthotopically transplanted a gene-edited pig kidney into a cerebral death patient.^6^ The transplanted kidney functioned successfully after operation, no hyperacute rejection was observed, and no chimeric or transmission of porcine retrovirus was detected. On January 7, 2022, the University of Maryland School of Medicine conducted the first clinical trial of cardiac xenotransplantation. They transplanted a pig heart edited with 10 specified genes to a 57-year-old patient with end-stage heart disease. Sadly, the patient died 2 months after the transplant.^7^

Organ transplantation is a significant breakthrough in the history of modern medicine, bringing new hope to patients with end-stage diseases. Although human beings have solved the technical problems of organ transplantation on the road of exploring organ transplantation, the problems of rejection, especially the initiation and maintenance of rejection still need to be solved.

## The danger model: immune response mediated by damage-associated molecular patterns (DAMPs)

**Fig. 2:**
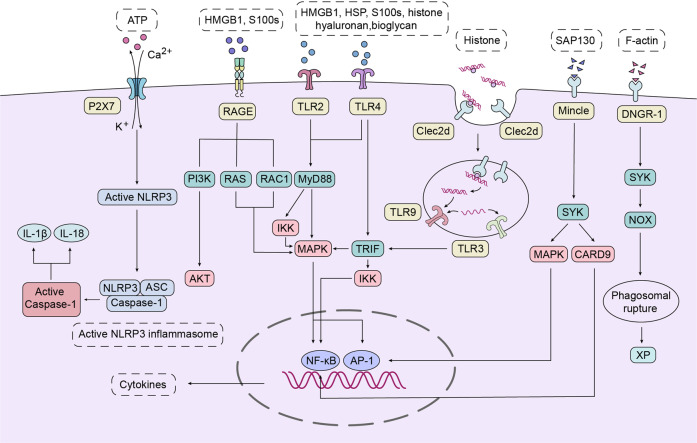
Receptors sense endogenous DAMPs activation intracellular signals. Receptors sense endogenous DAMPs activation intracellular signals. TLRs: TLRs can sense a variety of DAMPs. TLR2 and TLR4 recognize various DAMPs such as HMGB1, HSP, S100 protein, Histone, Hyaluronan, and Biglycan. Activated TLR2 and TLR4 recruit MAPK and IKK signals through cytoplasmic adapter MyD88. Results in activation of transcription factors NF-κB and activator protein 1 (AP-1), which mediate cytokine transcription. In addition to the MyD88 pathway, TLR4 also activates MAPK and IKK through TRIF. TLR3 and TLR9 are activated by endosomal nucleic acid. Like TLR2 and TLR4, TLR9 activated downstream signals through the MyD88 pathway, while TLR3 induced downstream signal activation through the TRIF pathway. CLRs: CLRs, a type of PRRs, contains multiple members, such as Clec2d, Mincle, and DNGR-1. Clec2d can be detected in cell membrane and endosomes. DNA-binding histones stimulate and activate endosomal TLR9 in a Clec2d-dependent manner, and induces inflammatory response. Mincle, another CLRs member, recognizes Sin3A-associated protein 130 (SAP130) and activates downstream MAPK signal through phosphorylation of spleen tyrosine kinase (SYK). SYK phosphorylation can also activate NF-κB through the assembly of caspase recruitment domain-containing protein 9 (CARD9) complex and promote the production of inflammatory factors. Unlike Clec2d and Mincle, DNGR-1 recognizes dead cell debris and promotes cross-presentation of associated antigens. DNGR-1 binds to the ligand filamentous actin(F-actin) to activate SYK. Phosphorylated SYK activated NADPH oxidase (NOX), causing the phagosome damage, and the phagosome contents to escape into the cytoplasm and access the endogenous major histocompatibility complex I antigen processing pathway, enabling MHC class I antigen to present exogenous antigens, thereby promoting the cross presentation of dendritic cells.^283^ RAGE: RAGE interacts with HMGB1 and S100s. Stimulated RAGE activates NF-κB and AP-1 through PI3K/AKT and MAPK pathways, promoting inflammatory response. P2X7: P2X7 receptors are activated by ATP to form non-selective ion channels that promote K^+^ efflux and Ca^2+^ influx. K^+^ efflux is critical for activation of NLRP3 inflammasome, which cuts pro-caspase-1 to form active caspase-1 and promotes IL-1β and IL-18 production. In addition, there is evidence that Ca^2+^ influx is involved in NLRP3 inflammasome activation

At the early stage of transplantation, surgically organ acquisition and implantation leads to ischemia-reperfusion. This process promotes the release of DAMPs that activate the immune cells as danger signals and play an important role in the activation and regulation of innate and adaptive immunity. This is formulated the danger model in immunology. In the danger model, the immune system recognize any form of cell stress/tissue injury rather than the presence of non-self. DAMPs are endogenous molecules which are passively or actively released under various conditions of major cell stress or tissue injury. In analogy to pathogen-associated molecular patterns (PAMPs), DAMPs are sensed by pattern recognition receptors (PRRs) on the surface or within the innate immune cells, as summarized in Table 1. Upon sensing of DAMPs, PRRs recruit MAPK, IKK signals through cytoplasmic adapter MyD88, phosphorylated SYK activated NADPH oxidase (NOX), or form non-selective ion channels that promote K^+^ efflux and Ca2^+^ influx (shown in Fig. 2).

**Fig. 3:**
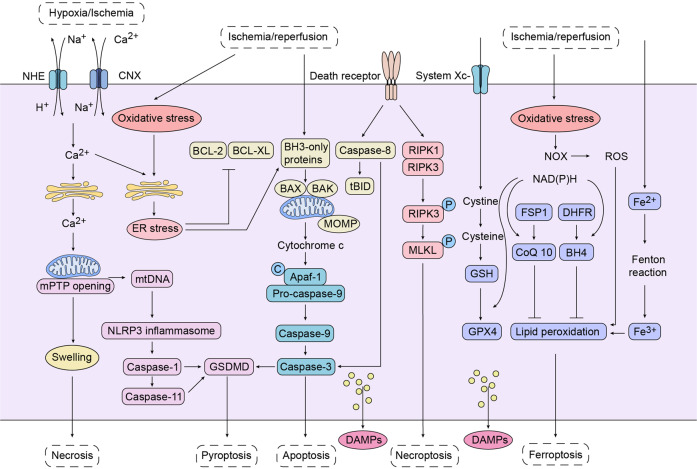
Ischemia and reperfusion mediate multiple pathways of cell death. Ischemia and reperfusion mediate multiple pathways of cell death. Necrosis: hypoxia/ischemia induces glycolysis and activates plasma membrane sodium proton exchangers (NHE) to promote Na^+^-H^+^ exchange, thus increasing intracellular Na^+^. Increased Ca^2+^ concentration in the mitochondrial matrix promotes rupture of the outer mitochondrial membrane (OMM) and ultimately necrosis. Pyroptosis: activated Caspase-1 hydrolyzes GSDMD to form functional GSDMD, which then aggregates in the plasma membrane to form pores. Mature IL-1β and IL-18 can be diffused into extracellular cells through this pore. Apoptosis: the triggering of apoptosis includes mitochondrial pathway and death receptor pathway. Bax and Bak polymerize to form a mitochondrial transmembrane pore, which induces the permeabilization of mitochondrial outer membrane (MOMP) and promotes the release of apoptosis-related soluble proteins, such as cytochrome c. Cytochrome c binds Apaf-1 to promote the formation of apoptosome, which in turn activates caspase-9 and its downstream caspase-3, triggering cell apoptosis.^284^ Necroptosis: binding of the death receptor and ligand promotes the interaction of RIPK1 and RIPK3, resulting in phosphorylation of PIPK3 and subsequent phosphorylation of the pseudokinase MLKL. Phosphorylated MLKL translocates to the plasma membrane, leading to membrane infiltration and triggering necroptosis. Ferroptosis: three cellular pathways are known to inhibit ferroptosis mediated by lipid peroxidation, including GSH/GPX4, FSP1/CoQ10, and DHFR/BH4. These three pathways require the participation of NAD(P)H. NADPH is the substrate of NADPH oxidase (NOX). Activated NOX positively regulates ferroptosis through production of ROS. Fe^2+^ overload could produce Fe^3+^ by Fenton reaction. Ferroptosis is triggered by Fe^3+^ through activation of lipoxygenase or inactivation of glutathione peroxidase (GPX4). GPX4 requires the participation of System X_c_^-^. System X_c_^-^ is a cystine/glutamate antiporter that promotes the entry of Cystine into cells. Cystine is then converted to cysteine, which produces glutathione (GSH). GSH is a cofactor of GPX4, which produces GPX4 with the participation of NADPH

Ischemia-reperfusion and mechanical injury accompanying transplantation lead to changes in the cellular microenvironment, which in turn mediate intracellular metabolic reprogramming. As shown in Fig. 3, multiple dramatic changes in cellular metabolism lead to the initiation of cellular stress or cell death pathways, and alterations in the intracellular transcription and expression and intracellular localization of some molecules, such as HMGB1, ATP, CIRP, and HSP, which are described further below, make these molecules potential immune mediators. In case of cellular stress or cell death, DAMPs can be passively released by cell death or actively released outside the cell and function as DAMP-activated immune signals.

### Ischemia/hypothermia

The grafts preserved by perfusate are in hypoxic/ischemic environment. In response to the reduction of available oxygen in the environment, cells actively mobilize the adaptation program and induce the production of anoxic adaptation factors. HIF-1 is a widely studied oxygen-sensitive factor, which is an adaptation factor to hypoxia. Under normoxia conditions, HIF-1 is degraded by ubiquitination and proteasome pathway, which is related to the hydroxylation of HIF-1α induced by proline hydroxylases (PHDs) and inhibitor of HIF factor (FIH1). Hypoxia inhibits PHDs and FIH1 activity and stabilizes HIF-α. Undegraded HIF-α forms a dimer with HIF-β via nuclear translocation, recruits and binds to p300 costimulatory molecules. Then positioned to hypoxia response elements (HREs) to regulate gene expression and regulate various physiological processes of cells to adapt to hypoxia.^8^ The stable expression of HIF-α is time- and temperature-dependent. Increased expression of HIF-α promotes apoptosis and activation of innate immunity.^9–11^ HIF-1 is also actively involved in cells metabolic reprogramming under hypoxia and hypo-nutrition.

During ischemia, cells exhibit increased glucose uptake, glycogen synthesis and glycolysis flux, as well as inhibition of mitochondrial metabolism and altered lipid metabolism. Increased cellular glucose uptake is associated with hypoxia-induced HIF-1, which regulates glucose transporter 1 (GLUT1) expression.^12^ The transcription of enzymes that regulate glycolysis by HIF-1 results in cells turning to anaerobic glycolysis due to reduced oxygen availability,^13^ then leads to lactate accumulation. Lactate in the environment is involved in regulating the function of innate and adaptive immune cells, which, to some extent, contributes to cell survival.^14^ However, the accumulation of lactate-H^+^ induces cell acidification and activates plasma membrane sodium proton exchanger 1 (NHE1).^12,15^ Activated NHE1 increases intracellular Na^+^ through Na^+^-H^+^ exchange, which is mediated by calcium-sodium exchanger (CNX), leading to Ca^2+^ overload and cell death.^16^ The inhibition of tricarboxylic acid cycle and electron transport chain is also an important metabolic event in mitochondria during hypoxia. This leads to ATP depletion and accumulation of tricarboxylic acid cycle metabolites such as succinic acid.^17^ ATP depletion inhibits Na^+^-K^+^ ATPase activity and leads to accumulation of intracellular sodium ions. High intracellular osmotic pressure leads to cell swelling and necrosis. Accumulated succinate regulates IL-1β expression by stabilizing HIF-1α.^18,19^ IL-1 β acts as a pro-inflammatory cytokine that triggers inflammation. In addition, sustained hypoxia inhibits the activity of the electron transport chain (ETC), resulting in excessive ROS production and cytotoxicity.^12^ Hypoxia also inhibits Stearoyl-CoA desaturase (SCD) activity in lipid metabolism.^20^ SCD is a rate-limiting enzyme for the synthesis of monounsaturated fatty acids.^21^ The limitation of SCD enzyme activity causes the accumulation of saturated fatty acids. On the one hand, it participates in endoplasmic reticulum stress and apoptosis.^12^ on the other hand, it can activate innate immune response through TLR2 and TLR4.^22,23^ In brief, hypoxia/ischemia-induced cellular metabolic reprogramming leads to the accumulation of intracellular metabolites such as succinic acid and saturated fatty acids involved in regulating the activation of innate immune responses. At the same time, dysregulation of cell metabolism can induce cell death. Some molecules isolated in cells are released upon cell death as DAMPs that are recognized by PRRs and participate in immune signal activation. The imbalance of cell metabolism can also cause endoplasmic reticulum stress response.

As previously mentioned, hypoxia/ischemia can cause disruptions in cellular calcium homeostasis, redox status balance and nutrient supply imbalance. The proper functioning of the endoplasmic reticulum depends on maintaining these balance to ensure proper protein folding and assembly.^24^ When these balance was destroyed, unfolded or misfolded proteins accumulated in the endoplasmic reticulum, inducing unfolded protein response (UPR), ER-associated degradation (ERAD), autophagy, hypoxic signaling transduction and mitochondrial biogenesis to maintain cell homeostasis. However, the persistence of endoplasmic reticulum stress will initiate the cell death pathway.^25,26^ The orderly process of intracellular metabolism requires continuous energy production. However, hypoxia/ischemia affects a variety of cellular metabolic pathways, resulting in reduced cellular energy supply and subsequent cellular stress. To a certain extent, this is a compensatory self-protection of cells. But long-term exposure to adverse conditions could cause injury.

In order to reduce the metabolic response of organs during in vitro preservation, a hypothermic preservation manner is derived to decrease the oxygen consumption of organs and cellular metabolic requirements.^27^ Hypothermia therapy has been proved to protect the damage caused by ischemia in multiple organs, such as the heart,^28^ liver,^29^ kidney^30^ and intestine.^31^ Static hypothermic preservation has long been used in clinical organ transplantation because of its strong operability. Although hypothermic preservation reduces graft injury to some extent, sustained hypothermia and rapid rewarming from hypothermia induce oxidative stress and cells injury.^32^

### Reperfusion/rewarming

Ischemia leads to metabolic changes and the production of immune mediators in the graft, which seriously damages the function and structural integrity of cells. The subsequent blood reperfusion/rewarming further aggravates ischemic injury, resulting in the eruption of reactive oxygen species (ROS) and inducing oxidative stress injury.^33,34^ The release of intracellular danger signals, including DAMPs, induces local infiltration of immune cells, fosters congenital and adaptive immune responses and further aggravates graft destruction.^34,35^ Vascular endothelial cells are damaged by various stress factors in reperfusion. The expression of activated endothelial cells up-regulated the expression of relevant DAMPs receptors, transmitted activation signals into the cell, promoted the secretion of inflammation-related cytokines and chemokines, and induced chemotaxis and infiltration of inflammatory neutrophils.^36–39^ Immune cells infiltrate into injured tissues, respond to the dangerous signals released by cells, and mediate the occurrence of inflammatory cascades and the clearance of damaged cells, in which the injury-related molecular patterns play a crucial role.

### Passive release of DAMPs from dead cells

The final state of cellular homeostasis disruption is cell death. In a way, the elimination of dead cells by the immune system is an effective way to maintain homeostasis. But a sustained or strong immune response can cause tissue and cell damage. However, cell necrosis, pyroptosis and ferroptosis lead to the destruction of membrane integrity and the release of cell contents, including various types of DAMPs, which are recognized and captured by immune cells and have significant pro-inflammatory effects. Different from the above cell death types, apoptosis is considered to be a cell death trans to immune silencing, in which ATP actively released by apoptosis acts as a find me signal and is quickly cleared by phagocytes. DAMPs exposed or released by apoptosis can effectively stimulate immune cells and induce inflammatory and adaptive immune responses under secondary death or specific stress stimulation. The following section focus on describing the above types of cell death.

## Apoptosis

Apoptosis, as a type of programmed cell death, is characterized by the formation of membrane-enclosed apoptotic bodies containing intracellular components. It is generally believed that cellular components, including DAMPs, are isolated in the membrane, and are rapidly taken up by phagocytes or adjacent cells after apoptosis occurs, without exposure of cell contents and producing no inflammatory response. Moreover, apoptosis stimulates macrophages to release anti-inflammatory cytokines IL-10 and TGF-β, which is a non-immunogenic cell death pathway to immune tolerance or immune silencing.^40–42^ The activation of cysteine proteases, or caspases, is the central link in cell apoptosis and mainly involves two pathways: intracellular mitochondrial pathway and extracellular death receptor pathway (Fig. 3C). These two pathways include the process of caspases being cleaved from the precursor pro-caspase to the activated caspase. Caspase exists in the form of inactive pro-caspase when it is not cut, which mainly includes three types. That is, initiator pro-caspases (human pro-caspase-8, -9 and -10), effector pro-caspases (human pro-caspase-3, -6, and -7) and inflammatory pro-caspases (human pro-caspase-1, -4 and -5).^40^ Initiator Procaspase and Effector procaspases are activated in sequence to form activated Effector caspases. Effector caspases subsequently cleaved multiple substrates to produce an apoptotic phenotype. Inflammatory pro-caspases play the role of inducing pyroptosis, which will be explained later.^40,43^ Diverse stimuli (e.g., calcium overload, endoplasmic reticulum stress) induce mitochondrial pathway mediated apoptosis, which is regulated by the BCL-2 family. The central event of mitochondrial apoptosis is mitochondrial outer membrane permeabilization (MOMP). MOMP releases soluble proteins such as cytochrome c from the mitochondrial intermembrane space, which binds to the adaptor molecule apoptotic peptidase activating factor 1 (APAF1) to form a complex of apoptosome. The complex then binding and activating pro-caspase 9 and cleaves and activates pro-caspase-3 and pro-caspase-7.^44^ Another pathway that induces apoptosis is mediated by death receptors. TNF-α is secreted by immune cells (macrophages, T lymphocytes) activated by inflammatory mediators and allogenic antigens exposed by the graft. TNF-α could bind to the death receptor TNF receptor 1 (TNFR1) on target cells to mediate downstream pro-caspase-8 activation. In addition, cytotoxic T lymphocytes activate target cells pro-caspase-8 via the Fas (another death receptor)/FasL pathway and subsequently activate downstream effector pro-caspases to mediate apoptosis.^43^ Although apoptosis has traditionally been thought not to cause inflammatory responses, there is evidence that Fas-mediated apoptosis stimulates intense inflammatory responses.^45^ Moreover, apoptosis induced by some cytotoxic chemotherapeutic (three anthracyclines and oxaliplatin) is immunogenic. This immunogenic cell death (ICD) was characterized by surface-exposed calreticulin (CRT), secreted ATP and released high mobility group protein B1 (HMGB1).^46^ These DAMPs are very important for ICD. Endoplasmic reticulum stress mediated CRT was exposed to the surface before apoptosis, which was phagocytosed by dendritic cells and assisted in antigen presentation, leading to specific cytotoxic T lymphocyte (CTL) response. ATP is secreted in a caspase-dependent manner in the early stage of apoptosis through a variety of pathways. As a ‘find me’ signal, ATP is recognized by P2Y2 receptor and attracts phagocytes. ATP released by dead cells activates the P2X7 receptor of dendritic cells, which activates the NALP3 inflammasome and drives IL-1β secretion, which assists in the production of CD8^+^T cells involved in adaptive immune responses. The interaction between extracellular HMGB1 and TLR4 correlates with ICD, and the depletion of TLR4 and the depletion or neutralization of HMGB1 can eliminate the cross-presentation of tumor antigens by DCs. But other studies have shown that HMGB1 released by apoptosis does not have immune-stimulating activity because apoptosis leads to the formation of oxidative HMGB1 via a caspase-dependent mechanism. Oxidized HMGB1 does not stimulate the production of pro-inflammatory cytokines, thereby promoting apoptosis-related tolerance. However, HMGB1 passive released by necrosis stimulates inflammatory response. Apoptosis can lead to secondary necrosis, in which the cell membrane allows the infiltration of macromolecules and the release of cell contents such as HMGB1.^41,46,47^ In conclusion, different from other cell death types described in this review, the plasma membrane of early apoptotic cells remains intact, and some DAMPs (such as ATP) can be actively released. Normally, DAMPs released at this time do not cause immune stimulation, and their main purpose is to be detected by phagocytes and then cleared quickly. However, secondary necrosis or middle and late apoptosis caused by late apoptosis can passively release DAMPs and cause inflammation. Moreover, DAMPs released by apoptotic cells are immunogenic, but occur under certain stress stimulation (such as the application of some chemotherapy drugs), and the apoptotic cells expose CRT, secrete ATP and release HMGB1, resulting in the death of immunogenic cells.

## Necrosis (Necroptosis)

Necrosis is generally considered to be the cell death that occurs as a result of intense physical or chemical stimulation and is characterized by cell swelling and destruction of cell membrane integrity (Fig. 3A).^48^ Necrosis is a non-programmed form of cell death that can cause a strong inflammatory response. This is associated with the release of various cellular contents. DAMPs released by necrosis include ATP, HMGB1, URIC acid, RNA and DNA. These DAMPs activate immune cells and induce production of pro-inflammatory cytokines.^49^ DAMPs released by necrosis can also serve as an endogenous adjuvant to aid in adaptive immune responses.^50^ This is of great significance for transplant rejection. With the deepening understanding of various types of cell death, the concept of necroptosis was brought in. Although morphologically similar to necrosis, necroptosis is a regulable type of programmed cell death, which is different from necrosis. Necroptosis occurs with the activation of receptor-interacting protein kinase (RIPK). There are two types of RIPK associated with necroptosis: RIPK1 and RIPK3. The interaction between RIPK1 and RIPK3 activates RIPK3, and then phosphorylates mixed lineage kinase domain-like pseudokinase (MLKL) (Fig. 3D).^51^ MLKL then translocates to the cell membrane and binds to phosphatidylinositol phosphate (PIPs), causing imbalance of cell ion homeostasis and rupture of osmotic membrane.^52,53^ This results in the potential passive release of DAMPs.

## Pyroptosis

Pyroptosis is a type of programmed cell death characterized by the formation of plasma membrane pores, cell swelling, and associated leakage of cell contents.^54^ Pyroptosis is initially thought to be mediated by active caspase-1 and can be triggered by cellular stress response, such as ischemia and reperfusion that induced mitochondrial calcium overload and the explosion of oxygen free radicals promoted the opening of permeability transition pore (PTP). Mitochondrial DNA fragments oxidized by oxygen free radicals enter the cytoplasm through PTP,^55^ and the oxidized mtDNA directly binds and activates NLRP3.^56^ Activated NLRP3 binds to the adaptor protein ASC and pre-caspase-1 to form the inflammasome complex and activate caspase-1, which cleaves pre-interleukin to form active IL-1β and IL-18. Caspase-1 cleaves gasdermin D (GSDMD), and then forms the plasma Membrane pore, which causes cell swelling and osmotic dissolution (Fig. 3B).^55,57^ In addition to caspase-1, other inflammatory caspases could cause pyroptosis. The linker between amino-terminal gasdermin-N and carboxy-terminal gasdermin-C domains in GSDMD can be specifically cleaved by caspase-1 and LPS-induced activation of caspase-4/5/11, which is necessary for cell pyroptosis.^58^ It has been point that caspase-3, which mediates apoptosis, can also cleave GSDMD and induce cell pyroptosis.^59^ Pyrophoric cells end up with swelling and bursting. Some cellular contents, including DAMPs, are passively released and participate in immune activation.

## Ferroptosis

Ferroptosis is a type of cell death characterized by iron-dependent lipid peroxidation and accumulation of reactive oxygen species (ROS). Ferroptosis was originally defined in tumor cells. Ferroptosis is morphologically, biochemical, and genetically distinct from apoptosis, necrosis, and autophagy. The morphology of Ferroptosis showed shrunken mitochondria, swelling of organelles and cytoplasm, rupture of outer membrane.^60^Ferroptosis is a type of cell death characterized by iron-dependent Lipid peroxidation and ROS accumulation.^60,61^Glutathione peroxidase 4 (GPX4) is an important antioxidant enzyme,^62^ which inhibits cell ferroptosis by eliminating lipid peroxides with Glutathione. By inhibiting the activity of GPX4, lipid peroxides cannot be metabolized by the reduction reaction catalyzed by GPX4, and Fe^2+^ oxidizes lipids in a Fenton-like manner, producing a large number of ROS to cause DNA damage, protein denaturation, and lipid peroxidation (Fig. 3E).^63,64^ Ferroptosis also triggers inflammation, partly attributable to DAMPs release. For instance, HMGB1 released by Ferroptosis plays an important role in inducing inflammatory responses. Inhibition of Ferroptosis can reduce the release of HMGB1 and inhibit inflammation.^65,66^

### Active release of DAMPs from living or stress cells

DAMPs are released when allografts undergo injury such as IR, infection and rejection.^67^ Except the passive release of DAMPs by dead cells, living cells or stress cells can also release DAMPs actively.^68^ Although the specific mechanism of the active release of DAMPs has not been fully elucidated, there is some evidence that the active release of DAMPs involves multiple pathways. DAMPs, such as ATP, can be secreted out of the cells in the form of exocytosis through vesicle transport of the Golgi apparatus and endoplasmic reticulum.^69^However, some DAMPs cannot be secreted by the classical pathway due to lack of precursor peptides. Post-translative modification is an important mechanism for the transfer of some DAMPs from the nucleus to the cytoplasm. Stress stimulates the exocytosis of accumulated DAMPs in the cytoplasm, and extracellular vesicles (e.g., exosomes) and secretory lysosomes may be involved in this pathway. For instance, active release of HMGB1 is highly correlated with its acetylation. Acetylated HMGB1 is repositioned to the cytoplasm and released extracellular by secreting lysosomes.^70,71^ The transfer of CIRP from the nucleus to the cytoplasm requires modification by methylation or phosphorylation. CIRP was translocated to the cytoplasm of stress particles, and released to the extracellular through exosomes. There is evidence that CIRP or HSPs may be released outside the cell via lysosomal secretion.^68,72^ In addition, DAMPs can be actively released by NLRP3 inflammasome. For example, active release of HMGB1 from immune cells depends on NLRP3 inflammasome.^73^ Although active release of DAMPs involves multiple pathways, there are still some gaps in the specific transport mechanism.

### Damage-associated molecular patterns (DAMPs)

Individual recognition and elimination of danger signals is a crucial ability acquired by individual immune system during evolution. During ischemia, in response to the intracellular stress response, some molecules are up-regulated in the cells to compensate for the intracellular metabolic imbalance before being recognized as DAMPs. When the integrity of the cell membrane is not broken, these molecules are isolated in the cells. Ischemia and reperfusion results in the exposure or release of DAMPs from the graft under stress. The recipient’s circulating immune cells recognize DAMPs with the aim of removing dead cells and repairing damaged tissue. However, the binding of DAMPs with immune cell receptors (PRRs) activates immune cells, resulting in a series of subsequent inflammatory cascade reactions (Fig. 2). At present, many DAMPs have been characterized at the molecular level, and they are vital in transplantation. The binding of DAMPs to relevant receptors is described in Table 1. In the following part, we discuss the release of some important DAMPs and their interaction with receptors to activate immune signals (Table 2).

**Table 1:** Damage-associated molecular patterns in sterile inflammation

Signal	Putative sensor	Release mechanism	Refs.
HMGB1	TLR2, TLR4, TLR9, RAGE, CD24, and TIM-3	Active release, passive release	^94,95,285–287^
HSPs	TLR2, TLR4, CD91, CD24, CD14, and CD40	Active release, passive release	^151,288,289^
S100 proteins	RAGE, TLR2, and TLR4	Active release, passive release	^80,290^
SAP130	CLEC4E (MINCLE)	Passive release	^291^
Histone	CLEC2D, TLR2, TLR4, and TLR9	Active release, passive release	^152,155,157,292^
RNA	TLR2, TLR3, TLR7, and TLR8	Passive release	^293,294^
DNA	RAGE, TLR9, and AIM2	Passive release	^87,295,296^
Lipids	CD300	Passive release	^297^
CIRP	TLR4/MD2	Active release, passive release	^68,72^
ATP	NLRP3, P2X, and P2Y	Active release, passive release	^110,120,298^
Hyaluronan	TLR2, TLR4, and CD44	Active release, passive release	^299,300^
Biglycan	TLR2, TLR4, and CD14	Passive release	^301,302^
Versican	TLR2 and CD44	Passive release	^303^
Heparan sulphate	TLR4	Passive release	^304^
Formyl peptides (mitochondrial)	FPR1	Passive release	^305^
DNA (mitochondrial)	TLR9 and NLRP3	Passive release	^306^
CPPD crystals	NLRP3	Passive release	^307^
β-amyloid	NLRP3, CD36, and RAGE	Passive release	^308–310^
Cholesterol crystals	NLRP3 and CD36	Passive release	^311,312^
Uric acid/MSU	NLRP3	Passive release	^307,313^
IL-1α	IL-1R	Passive release	^314^
IL-33	ST2	Passive release	^315^
BCL-2	TLR2	Passive release	^316^
Silica	NLRP3	Passive release	^317^
Asbestos	NLRP3	Passive release	^318^
Filamentous actin	CLEC9A(DNGR-1)	Passive release	^319^

**Table 2:** Clinical trials and FDA-approved targeted drugs

Target	Drugs	Mechanism	Drug type	Indication	Research status
DNA	Azathioprine	Inhibition of DNA synthesis	Small molecule	Kidney, heart, liver, lung transplantation	GP
Calcineurin	Tacrolimus Cyclosporine	calcineurin inhibitor	Small molecule	Organ transplantation	GP
mTOR	Sirolimus	mTOR inhibitor	Small molecule	Kidney transplantation	GP
mTORC1mTORC2	Everolimus	mTORC1, mTORC2 inhibitor	Small molecule	Kidney, heart, liver transplantation	GP
Cytokines	Deoxyspergualin hydrochloride	Cytokines inhibitor	Small molecule	Kidney transplantation	GP
GR	Prednisolone Meprednisone	GR stimulant	Small molecule	Organ transplantation	GP
IMPDH	MPS, MFF, Mizoribine	IMPDH inhibitor	Small molecule	Kidney, heart, liver, lung transplantation	GP
CD86 CD80	Belatacept	CD86, CD80 inhibitor	Fusion protein	Kidney transplantation Lung transplantation	GP Stage II
IL-12, STAT4,SSTR4, LPAAT	Lisofylline	IL-12, STAT4, SSTR4, LPAAT inhibitor	Small molecule	Organ transplantation	Stage III
EDG1	Mocravimod dihydrochloride	EDG1 inhibitor	Small molecule	Organ transplantation	Stage II
PPARα PPARγ	Saroglitazar	PPARα, PPARγ agonist	Small molecule	Liver transplantation	Stage II
CXCR1 CXCR2	Reparixin	CXCR1,CXCR2 antagonist	Small molecule	Liver transplantation	Stage II
Cysteine proteinase	Imlifidase	Cysteine protease stimulant	Synthetic peptide	Kidney transplantation Organ transplantation	GP PMA
T cells	ATG-Fresenius S	Immunosuppression	Ig	Organ transplantation	GP
Thymic lymphocytes	ATG	Immunosuppression	Ig	Organ transplantation	GP
CD20	Rituximab	CD20 inhibitor	mAb	Kidney, liver transplantation	GP
CD3	Muromonab-CD3	CD3 inhibitor	mAb	Liver, heart transplantation	GP
CD5	Olendalizumab	CD5 inhibitor	mAb	Organ transplantation	Stage II
CD19	Inebilizumab-cdon	CD19 inhibitor	mAb	Kidney transplantation	Stage II
CD40	Bleselumab Iscalimab	CD40 inhibitor	mAb	Organ transplantation	Stage II
CD40L	Tegoprubart	CD40L inhibitor	mAb	Organ transplantation	Stage II
CD28	Lulizumab pegol	CD28 inhibitor	mAb	Kidney transplantation	Stage I/II
CD38	Isatuximab-IRFC	CD38 inhibitor	mAb	Kidney transplantation	Stage I/II
IL2RA	Basiliximab,Daclizumab	IL2RA inhibitor	mAb	Organ transplantation	GP
IL-6	Clazakizumab	IL-6 inhibitor	mAb	Kidney transplantation	Stage III
IL-18	GSK-1070806	IL-18 inhibitor	mAb	DGF	Stage II
IFN-γ	Emapalumab-LZSG	IFN-γinhibitor	mAb	Organ transplantation	Stage II
ITGAL	Odulimomab	ITGAL antagonist	mAb	Kidney transplantation	GP
BLyS	Belimumab	BLyS inhibitor	mAb	Kidney transplantation GVHD	Stage II Stage I
C1s	BIVV-020	C1s inhibitor	Small molecule	Kidney transplantation	Stage II
C3	Pegcetacoplan	C3 inhibitor	Synthetic peptide	Kidney transplantation	Stage I
HLA-A2	TX-200 QEL-001	HLA-A2 inhibito	CAR-T	Organ transplantation Liver transplantation	Stage II Stage I/II

## HMGB1

High-mobility group box 1 protein (HMGB1) is a nuclear non-histone chromatin binding protein involved in transcriptional regulation and is expressed in almost all nucleated cells.^46^ The release of HMGB1 from cells induced by ischemia and reperfusion can be passively released by cell death and actively released by cell stress response. The active release of HMGB1 is associated with its high acetylation level.^70^In the nucleus, histone acetyl transferase (HATs) regulates the hyperacetylation of nuclear proteins, including HMGB1, while the activity of Histone deacetylation enzymes (HDACs) can achieve an equilibrium state of acetylation.^74^ Histone deacetylases (HDACs) act by modulating HMGB1 deacetylation during cell homeostasis. When oxidative stress occurs, the activity of HDACs is inhibited, which is closely related to the acetylation of HMGB1. Studies have shown that low expression of HDACs1 and HDACs4 promotes the translocation and release of HMGB1.^75^ HMGB1 is also regarded as a cytokine released by activated monocytes/macrophages with delayed release. Monocytes/macrophages release pro-inflammatory cytokines such as TNF and IL-1 early and HMGB1 later. Due to lack of lead peptide sequence, HMGB1 could not be secreted into the extracellular space through the classical endoplasmic reticulum-Golgi extracellular pathway.^70^ Extensive acetylation of HMGB1 in activated monocytes/macrophages relocates HMGB1 to the cytoplasm, and the acetylation of HMGB1 is transferred to the secretory endolysosomal compartment and released extracellular.^71,76^ HMGB1 can also be actively released due to methylation. A research described the release of methylated HMGB1 from the nucleus and extracellular of neutrophils. Lys42 of HMGB1 is post-translationally and methylated at the end of neutrophil differentiation in myelocytic cells. The methylation of Lys42 of HMGB1 enables the localization of HMGB1 to the cytoplasm. The diffusion of methylated HMGB1 from the nucleus to the cytoplasm is due to the decreased affinity for chromosomal DNA. When neutrophils are stimulated by activation signals, HMGB1 in the cytoplasm can be released extracellular. However, how HMGB1 methylation occurs and is released from cells has not been clarified.^77^

The released HMGB1 binds to RAGE and is involved in regulating innate and adaptive immune responses. The receptor for advanced glycation end products (RAGE) is the first receptor identified as binding to HMGB1. It is a transmembrane protein of the immunoglobulin (Ig) superfamily, which contains three extracellular Ig like domains that recognize and bind ligands, and is expressed in a variety of cells, including endothelial cells, macrophages, dendritic cells, and T cells.^78,79^ RAGE actively participated in the activation of immune response during organ transplantation. Under normal physiological conditions, the expression of RAGE was generally low.^80^ When organs undergo ischemia/reperfusion, the expression of RAGE in vascular endothelial cells is up-regulated.^81,82^ IRI induces the release of DAMPs from various RAGE ligands, including HMGB1, S100 proteins and DNA. These ligands bind and activate RAGE on endothelial cells, and induce NF-κB nuclear internalization through MAPK signaling pathway, regulate the expression of target genes, and release cytokines and chemokines.^38^ Activated endothelial cells could produce more adhesion molecules ICAM-1 and VCAM-1 through NF-κB, and promote the adhesion of immune cells.^38,83^ The binding of RAGE and HMGB1 is involved in transforming blood vessels into a high permeability state, which allows immune cells to infiltrate injury tissues.^84,85^ Soluble RAGE treatment reduced endothelial barrier permeability by disrupting the crosstalk between HMGB1 and RAGE.^86^ HMGB1 or DNA can bind to RAGE alone to induce sterile inflammation. RAGE mediated DNA uptake through the endosomal pathway and reduced the immune recognition threshold of TLR9. TLR9 is a DNA sensor, which is described below. Interestingly, the HMGB1-DNA complex was more effective in activating plasmacytoid dendritic cells than HMGB1 alone.^87^ The combination of HMGB1 and RAGE has tissue specificity in inducing inflammatory response in organ ischemia-reperfusion. For instance, in mice heart and liver ischemia/reperfusion models, HMGB1 bind to RAGE increase activation of the proinflammatory MAPK signaling pathway and nuclear translocation of NF-κB, stimulates the expression of inflammatory cytokines such as TNF-α and IL-6, and induces tissue cells inflammation injury.^88–90^ However, in a renal ischemia/reperfusion model, although the expressions of HMGB1 were detected to be elevated, the inflammatory response was inhibited and kidney injury was alleviated after blocking HMGB1. The loss of RAGE had no significant effect on renal IRI. These results suggest that HMGB1 may induce inflammatory response in renal IRI by binding other receptors (such as TLR2 and TLR4) rather than RAGE.^91^ Some evidence suggests that HMGB1/RAGE is involved in adaptive immune. Activated TLR9 stimulates DCs to actively secrete HMGB1. HMGB1 binds to RAGE expressed on DCs, and this autocrine mode activates MAPKs and NF-κB signals, then stimulates DCs to secrete IL-2 and maturation.^92^ The activation and proliferation of T cells require RAGE, but the initiation of T cells is not directly generated by the binding of RAGE expressed on T cells to ligands.^93^ A study showed that HMGB1 binds to CD103^+^DCs with high expression of CD24, forming a membrane CD24-HMGB1 conjugate and binds to the RAGE expressed on CD8^+^T cells. This CD24-HMGB1-RAGE axis stimulates the activation of CD8^+^T cells and secretes IL-2.^94^

HMGB1 also mediate pro-inflammatory effects by binding to TLRs, including TLR2, TLR4, and TLR9.^95^ These receptors are composed of ligand-bound N-terminal leucine-rich repeat signal domain, transmembrane domain and intracytoplasmic tail composed of Toll/IL-1R homologous domain.^96^ TLRs are expressed in innate immune cells, such as monocytes, macrophages, dendritic cells, neutrophils, and natural killer cells.^97^ When HMGB1 bind to TLR2, it triggers the activation of the downstream cell signaling adapter MyD88 and induces NF-κB signal activation.^98^ TLR2 is associated with early graft injury. For instance, TLR2 and RAGE are involved in HMGB1-mediated early islet loss in mice islet transplantation, rather than TLR4. Islets are rich in HMGB1, and the release of HMGB1 from islet cell injury stimulates up-regulated CD40 expression and IL-12 secretion in DCs, resulting in IFN-γ production by NKT cells and GR-1^+^CD11b^+^cells.^99^ TLR4 is activated and upregulated in ischemia/reperfusion injury.^100^ HMGB1 binds TLR4 to induce inflammation. Activation of TLR4 mediates the production and release of cytokines and chemokines.^97^ TLR4 signals trigger both MYD88 and TRIF pathways. This results in the translocation of NF-κB from the cytoplasm to the nucleus, inducing production of pro-inflammatory factors such as IL-6 and TNF, and chemokine CCL2. Activation of the TLRs/MYD88 pathway promotes transcription of inflammatory cytokine precursors pro-IL-1β and pro-IL-18 and assembly of inflammasome.^96^ However, pretreatment of nonharmful quantity of recombinant HMGB1 (rHMGB1) induces increased expression of Sialic acid-binding Ig-like Lectins (siglec-10, a negative regulator). HMGB1 binds to CD24 and interacts with Siglec-10, inhibiting the NF-κB nuclear translocation induced by TLR4 activation, resulting in a decrease in the expression of IL-6, TNF-α, CXCL2 and CCL2.^101^ As mentioned above, TLR9 is a DNA sensor that can recognize microbial DNA and endogenous DNA.^102,103^ TLR9 is localized to the endoplasmic reticulum of immune cells (macrophages and dendritic cells). CpG DNA binds TLR9, causing TLR9 to redistribute from the endoplasmic reticulum to the endosome containing CpG DNA, and recruiting MyD88 to initiate signal transduction.^104^ HMGB1 can enhance the response of TLR9 to DNA, leading to increased secretion of IL-6, IL-12, and TNFα dependent on TLR9.^102^ The nuclear DNA-binding protein HMGB1 released by necrotic cells binds to extracellular DNA to form complex, which stimulates cytokine production of RAGE through TLR9-MyD88 pathway.^105^ In addition, HMGB1 and histones released by cell injury can stimulate the formation of neutrophil extracellular traps (NETs) through TLR4 and TLR9-MyD88 signals. NETs are extracellular scaffolded of nuclear DNA filled with granular proteins, histones and cytoplasmic antimicrobials that are thought to benefit host defense against pathogenic microorganisms. However, NETs are considered to be deleterious in a variety of sterile inflammation, including atherosclerosis, vasculitis, thrombosis, systemic lupus erythematosus (SLE), rheumatoid arthritis (RA), lung injury, and tumor metastasis.^106,107^ In conclusion, HMGB1 is secreted extracellular as DAMPs or a cytokine of immune cells, mediates the recruitment and infiltration of immune cells via binding to receptors, stimulates and amplifies inflammatory signals, and indirectly stimulates the activation of innate cells.

## ATP

Adenosine triphosphate (ATP) is a hub of intracellular energy supply. It releases energy upon hydrolysis and is called energy currency. In addition to intracellular energy supply, ATP released out of the cell is regarded as a danger signal molecule, which participates in the activation of the immune response during organ transplantation, mediates rejection reaction, and affects the short-term and long-term survival of the graft.^108^ Cell death and stress from ischemia promote the release of ATP into the extracellular space, which is exacerbated by reperfusion, although it is necessary for the graft.^109^ ATP is released out of the cell through a variety of pathways. During ischemia and reperfusion, in addition to passive release through membrane rupture, ATP can be actively released out of the cell through exocytosis (secreting lysosomes and extracellular vesicles) and membrane channels.^68,110^ To date, there are five major ATP release channels identified, namely connexin hemi-channel (connexin hemi-channel), Pannexin 1 (PANX1), Calcium homeostasis Modulator 1 (Calcium homeostasis Modulator 1), CALHM1), volume-regulated anion channels (VRACs), and Maxi-Anion channels (MACs).^111^ ATP and its metabolites (ADP, AMP, adenosine) released through the PANX1 channel provide autocrine and paracrine signals and play an important role in regulating the immune response. Extracellular ATP binds to P2X (ligand-gated ion channels) and P2Y receptors (Gq and Gi coupled receptors) of target cells, activating the PANX1 channel through positive feedback on the one hand and regulating the immune system on the other.^112^ ATP binds to P2X7R and activates immune cells such as macrophages and neutrophils to induce K^+^ efflux, which plays an important role in the activation of NLRP3.^113,114^ NLRP3 is a member of the nucleotide-binding oligomerization domain-like receptors (NLRs). NLRP3 inflammasome is composed of NLRP3, the adaptor protein ASC and pre-Caspase1. Activation of NLRP3 inflammasome is significant in inflammatory response. The activation process may be different in different cells, but in general, the activation of NLRP3 inflammatory cells has undergone two steps: transcription initiation and activation. The transcription initiation of NLRP3 stimulates the activation of NF-κB through PRRs, and NF-κB participates in the transcription of NLRP3.^115,116^ NLRP3 is stimulated and activated after transcription and translation to participate in the assembly of NLRP3 inflammatory bodies. Inactive state of NLRP3 before activation is partly due to ubiquitination of NLRP3.^115^ In a recent study, it was found that extracellular ATP can promote the deubiquitination of NLRP3 by inducing phosphorylation of Paxillin (Y118), which depends on extracellular ATP and K ^+^ efflux, and requires the participation of USP13. In addition, the presence of ATP induces the recruitment of Paxillin and NLRP3 to the cell membrane. And it interacts with P2X7R to form the P2X7R-Paxillin-NLRP3 complex, which in turn stimulates the assembly of NLRP3 inflammasomes.^117^ Different stimuli recruit NLRP3 to dTGN by binding to PtdIns4p on dTGN (the dispersed TGN (the trans-Golgi network)) through its polybase region, which depends on K ^+^ outflow. dTGN acts as a scaffold for NLRP3 aggregation and ASC binding to further activate downstream signals.^118^ Pro-caspase1 binds to NLRP3 and ASC to form activated Caspase1, which cleaves downstream Pro-IL-1 and Pro-IL-18.^115^ P2RX7 senses extracellular adenosine triphosphate (eATP) and is essential for recirculating CD8^+^ T cell memory. A recent study showed that P2X7R is critical for the generation of tissue-resident memory (Trm) CD8^+^ T cells. P2RX7 supports the generation of Trm cells by enhancing the perception of TGF-β by CD8^+^ T cells, and is beneficial to the long-term maintenance of Trm.^119^ ATP, however, is thought to inhibit inflammation at low doses or chronic exposure. Low concentrations of ATP inhibit macrophage production of IL-12 and TNF-α, which may depend on the P2Y receptor with high affinity for ATP.^120^ In addition, CD39 and CD73 are involved in the transition from an ATP-driven pro-inflammatory environment to an adenosine-induced anti-inflammatory environment. CD39 and CD73 catalyze the production of AMP from ATP and ADP, and CD73 converts AMP to adenosine. Adenosine can be returned to cells by adenosine transporters (Nucleoside transporters) or binding to P1 (A2A, A2B) receptors as a ligand. The activation of P1 receptor plays an anti-inflammatory role and inhibits adaptive immune response.^120,121^ In conclusion, ATP can have either pro-inflammatory or anti-inflammatory effects. To some extent, the pro-inflammatory effects of ATP depend on the production level of ATP and the receptors it binds to. The binding of ATP and P2X7R induces the production of pro-inflammatory cytokines. Low doses or chronic exposure of ATP depend on P2Y receptors to inhibit inflammation.

## S100 protein

The S100 protein is a calcium-binding protein family consisting of 25 members. S100 protein is only expressed in vertebrates and involved in regulating inflammation, cell differentiation, proliferation, energy metabolism, apoptosis, calcium homeostasis, cytoskeleton, and other pathways.^122^ Activation of S100 protein depends on the binding of metal ions (calcium, zinc, or copper) and the formation of homodimer or heterodimer.^123^ S100 participates in the regulation of inflammation in myocardial infarction, stroke, brain trauma, and atherosclerosis. And S100 protein is thought to be a biomarker indicates disease progression and prognosis in inflammation, allergy, heart disease, and cancer.^124^ S100 protein is passively released by dead cells and actively released into the extracellular space by stressed or activated macrophages and neutrophil cells. Like HMGB1, S100 protein does not have a leading peptide sequence and is not released into the extracellular domain through the classical pathway. It may be secreted extracellular by non-classical pathways.^125^ S100 protein was detected in exosomes and played a functional role. For example, exosomes present in follicular fluid contain the S100A9 protein. Exosomes enriched in S100A9 significantly enhance inflammatory response through activation of NF-κB signaling pathway.^126^ S100A8/A9 is actively delivered to the tumor microenvironment and distant tissues by exosomes secreted by myeloid-derived suppressor cells (MDSC).^127^ It suggests that the active release of S100 protein may be related to exosomes. But the specific release mechanism has not been fully elucidated. The extracellular space contains a high concentration of calcium. When S100 protein is released into the extracellular high calcium environment, it binds with calcium and forms a calcium-loaded state, which is the key to binding with RAGE. The activated RAGE can use S100 protein as the target gene and up-regulate the expression of S100 protein, forming a positive feedback loop and amplifying inflammation.^123,128^ S100A8/A9, and S100A12 bind to RAGE or TLR4 as pro-inflammatory ligands.^80^ S100A8 and S100A9 usually form heterodimers, and S100A8/A9 is highly expressed in the cytoplasm of neutrophils (40%) and monocytes (5%). It has been described that transcription of S100A8 and S100A9 can be induced by TNF-α or IL-1 β via C/EBPα. Additionally, HIF-1 has been demonstrated to increase S100A8/A9 mRNA levels in prostate cancer epithelial cells by directly binding to the S100A8 and S100A9 promoters.^129^ S100A8/A9 can be either passively released due to cell death or the formation of NETs, or actively released through inflammation. Extracellular S100A8/A9 interacts with TLR4 and RAGE to promote immune cells activation and recruitment. S100A8/A9 binds to TLR4 to activate β2 integrin and induce neutrophil recruitment. Moreover, S100A8/A9 upregulates the expression of monocyte CD11b and chemokines (such as CXCL10) to promote cell adhesion and chemotaxis. TLR4 mediates NF-κB nuclear transfer and further induces the expression of cytokines and chemokines through TIR-domain containing adaptor molecules TRAM/TRIF and MyD88.^130^ Unlike S100A8/A9, although S100A12 interacts with the TLR4 receptor of monocytes to trigger inflammatory signals, blocking the binding of S100A12 to RAGE had no significant effect on inflammatory signal transduction in monocytes expressing RAGE.^131^ The production of S100 protein increased in the early stage, which may be a potential biomarker in transplantation. In graft-versus-host disease, serum concentrations of S100A8/S100A9 and S100A12 are significantly elevated during the acute phase. The binding of S100A8, S100A9, S100A8/A9, or S100A12 with TLR4 leads to the activation of monocyte transcription factor NF-kB and the secretion of pro-inflammatory cytokines such as IL-1β, IL-6, IL-8, or TNF-α, thereby promoting the development of Th17 cells.^132^ As the most widely studied S100 proteins, S100A8, S100A9, and S100A12 act as DAMPs to interact with TLR4 or RAGE extracellular, mediating the recruitment and activation of immune cells and secreting pro-inflammatory cytokines. Moreover, these proteins can appear in serum at the early stage of transplantation and may be a potential biological marker.

## CIRP

Cold-inducible RNA-binding protein (CIRP), a member of the Cold shock protein family, is induced to express at low temperatures. There is a conserved RNA-recognition motif (RRM) at the N-terminal of CIRP, containing two ribonucleoprotein domains: RNP1 and RNP2, which belong to the GRP subfamily and may be involved in the post-transcriptional regulation of gene expression. There is a poorly conserved Glycine-rich domain (RGG) domain at c-terminal, which contains multiple ARG-Gly-Gly (GRR) repeats, which may be related to the cytoplasmic translocation of CIRP.^133,134^ Except to being induced by hypothermia, CIRP can also be induced by hypoxia/ischemia and reoxygenation/reperfusion, but the expression level of CIRP may be related to hypoxia/ischemia time. Studies have demonstrated that the expression level of CIRP in cardiomyocytes with hypoxia time of 72 h in vitro is reduced.^135^ An increased CIRP expression is essentially a benefit to the cell. Overexpressed CIRP can actively exert anti-apoptotic effects through multiple pathways. Increased expression of CIRP in TNF-α signaling pathway by inhibiting the activation of caspase-8, play the role of core death signaling inhibitor. At the same time, CIRP induces MAPK/ERK1/2 cascade pathway and NF-κB pathway, activates Bcl-2 and Bcl-xl to induce anti-apoptosis and inhibits Bax and Bad-activated caspase-9 apoptosis.^133^ However, the accumulation of CIRP during ischemia may result in its massive release into the extracellular domain during reperfusion. CIRP is passively released by cell death during reoxygenation (reperfusion) and actively released by stressed cells. CIRP cannot be secreted extracellular by the classical pathway. Under the conditions of er stress and oxidative stress, CIRP transfer from nucleus to cytoplasm requires post-translational modification (methylation and phosphorylation). CIRP transfers from nucleus to stress granules of cytoplasm, and is released into the extracellular through exosomes and other ways.^68^ CIRP was detected in lysosomes of RAW 264.7 cells after reoxygenation in vitro and co-located with lysosomal protein manufacturing protein cathespin D, suggesting that CIRP may be released into the extracellular through lysosomes.^72^ CIRP released to the extracellular space is identified as a DAMPs and participates in inflammatory response as a danger signal. The interaction between CIRP and immune cells receptors is mostly described in sepsis model, but it has not been reported in ischemia-reperfusion or transplantation model at present. CIRP binds to TLR4, myeloid differentiation Factor 2 (MD2) and TLR4-MD2 complex with a high affinity. In macrophages under hypoxia stress, CIRP is translocated from the nucleus to the cytoplasm and released. In vivo injection of recombinant CIRP stimulates macrophages to secret TNF-α and HMGB1, inducing an inflammatory response. And extracellular CIRP activity is mediated by TLR4-MD2 complex.^72^ CIRP treatment increases the ICAM-1^+^ phenotype of bone marrow derived neutrophils (BMDN) and stimulates the production of iNOS and NETs in a TLR4-dependent manner.^136^ CIRP stimulates macrophages to release pro-inflammatory cytokines in sepsis and plays an important role in T cell dysregulation. Recombinant CIRP treatment significantly increased the expression of CD69 and CD25 on CD4^+^ and CD8^+^ spleen T cells in TLR4-dependent manner. Moreover, CIRP treatment predisposed CD4^+^ T cells to Th1 hyperinflammatory response and affected the cytotoxicity of CD8^+^ T cells.^137^

## HSPs

Under stress conditions, cells produce a group of stress proteins, including heat shock proteins (HSPs), RNA chaperones and ER stress-related proteins, which play an important role in maintaining intracellular homeostasis.^138^ HSPs is a chaperone protein, which can be divided into macromolecule HSPs (100 kD), HSP90 (81–99 KD), HSP70 (65–80 KD), HSP60 (55–64 KD), HSP40 (35–54 KD), and micro-molecule HSPs (≤34 KD) according to molecular size.^139^ HSPs facilitates the natural folding and stabilization of newborn proteins in cells, and the intracellular environment of tissues subjected to ischemia/reperfusion changes. Under the stress environment such as high temperatures, toxins, oxidative conditions, and glucose deprivation, the expression of HSPs is up-regulated, mediating the folding of newborn peptides and the repair and decomposition of unfolded or misfolded proteins.^140,141^ Heat shock factors (HSFs) are activated by a variety of intracellular stress responses during ischemia/reperfusion. The activated HSFs bind to Heat shock elements (HSE) of HSPs, which is the promoter element of HSPs gene. May involve multiple Adjacent Inverted arrays of the binding site (5-NGAAN-3), facilitating transcription of HSPs.^142^ A research points that the overexpression of graft HSPs, however, induced faster rejection in the mouse skin graft model, but the mechanism is not explained.^143^ The increased intracellular HSPs during graft ischemia/reperfusion play an important role in the maintenance of intracellular homeostasis, but injury cells lead HSPs to be passively released out of the cells and identified as DAMPs, which are involved in several immune activation processes and are an important component of elicited immune response. The active release of HSPs can be mediated by extracellular vesicles, which can be divided into exosomes and micro-vesicles and also be secreted to the extracellular by lysosomal pathway.^68,144^ Extracellular HSPs induce NF-κB phosphorylation, up-regulate monocyte chemoattractant protein-1 (MCP-1) and intercellular adhesion molecule-1 (ICAM-1) production in endothelial cells, and stimulate macrophages to produce and secrete cytokines (TNF-α, IL-1β, and IL-6) through TLR2 and TLR4.^145–147^ CD91 is a signal receptor for HSPs (gp96, HSP70, HSP90, calreticulin) expressed on antigen presenting cells (APCs), allowing APC to secrete inflammatory cytokines such as TNF-α, IL-1β, IL-12, and granulocyte macrophage colony-stimulating factor (GM-CSF) via p38 MAPK and NF-κB pathways, and inducing macrophages and dendritic cells to produce inducible no-oxide synthase (iNOS) and nitric oxide, respectively. It should be noted that different HSPs stimulate distinct cytokine secretion profiles, which may be related to distinct phosphorylation sites of CD90 activation by HSPs.^148,149^ Different cytokine microenvironments induced by different HSPs determine the priming of specific T-helper cell subsets. For example, the TGF-β microenvironment induced by immunization with calreticulin can initiate Th17 cell response rather than gp96 or HSP70.^149^ CD91 plays another role in presenting antigenic peptides chaperoned by HSPs (such as gp96). The gp96 is only present in cells and is released by necrotic cells, so CD91 could acts as a sensor for necrotic cells to prime immunity.^150^ HSPs (gp96, HSP70, HSP90, calreticulin) bind to peptides to form HSP peptide complexes. HSPs protects peptides bound to them from degeneration and degradation. HSP-peptide complexes induce MHC-restricted antigen specific CD8^+^ cytotoxic T lymphocyte (CTL) responses. Exogenous antigens are usually presented by MHC Class II molecules of antigen-presenting cells (APCs). However, exogenous HSPs-peptides can be presented by endogenous MHC Class I molecules to initiate cross-priming, reflecting the powerful adjuvant properties of HSPs.^148^ Although some receptors trigger HSPs-induced activation of the immune system, there are also inhibitory receptors that mediate immune suppression. CD24 and Siglec-10 associate to negatively regulate the stimulatory activity of HSP70, HSP90, and HMGB1, and inhibit the activation of NF-κB to protect the host from the fatal response of pathological cell death.^151^ This may be a potential target to protect against graft immune damage.

## Histone

Histones are a class of highly basic proteins rich in lysine and arginine residues that are found in the nucleus, including H1/H5, H2A, H2B, H3, and H4, and are involved in gene regulation and DNA replication. They are released extracellular by injured cells during ischemia/reperfusion, sepsis, and autoimmune diseases.^152^ The mechanism of histone active release has not been fully elucidated. A study described that histones could be actively released by activated macrophages through soluble and extracellular vesicles.^153^ Histone aggravates cells damage via direct toxicity to cells and mediating inflammatory response. Here, we primarily discuss the role of histones as DAMPs recognized by immune cell receptors. Histones exist in the form of free and DNA binding. Extracellular histone increases leukocyte adhesion (neutrophils and monocytes) and microvascular permeability. Histone directly interacts with TLR2 and TLR4 to induce activation of MyD88, NF-κB, and MAPK signaling pathways and stimulate production of pro-inflammatory cytokines (IL-6 and TNF-α) and chemokines (CXCL2).^154^ Histones are recognized by TLR9 receptors of immune cells and mediate the production of IL-6 and TNF-α through the MyD88 pathway, exacerbating cells injury. And the damage was significantly reduced via neutralization of histones. On the other hand, extracellular histones enhance DNA-mediated TLR9 activation in immune cells through direct interaction.^155^ NLRP3 inflammasome also play an important role in histone induced inflammatory responses. Histones have been shown to induce IL-1β secretion in an NLRP3 inflammasome dependent manner.^156^ Moreover, a recent study identified CLEC2D expressed by macrophages as a sensor that can sense histones released by dead cells. The recognition of histones by CLEC2D depends on the positive charge on its tail lysine residue. CLEC2D can perceive histone-DNA complex and transfer it to the nucleosome to activate TLR9, causing TLR9-dependent cell signal activation. However, it is unclear how CLEC2D transfers histone complexes to the nucleosome.^157^

## The stranger model: immune response mediated by allo-antigens

Besides DAMPs, allo-antigens also trigger immune response to graft in recipients. The immune system can distinguish self and non-self and response to non-self, called the stranger model. Individual genetic differences between host and donor cause recognition of non-self. Not only the host, but also the residual immune cells in the allograft could recognize the alloantigens and cause graft-versus-host disease. It is mainly concerned in allogeneic hematopoietic stem cell transplantation (AHSCT). In solid organ transplant, ‘non-self’ antigen of major histocompatibility (MHC) receptors are innate and adaptive immune cells express recognition, including induction of T and B lymphocytes and innate immune cells of graft rejection.

## xMHC

All along, the existence of allogeneic antigens is the key to the rejection of allogeneic organ transplantation. The recognition of allogeneic ‘non-self’ antigens by recipient immune cells stimulates a series of immune responses and immune attacks on the transplanted organ. The major histocompatibility complex (MHC) is the most important gene cluster in organ transplantation. The human MHC region is located on the short arm of chromosome 6 (6p21.3) and contains about 250 gene loci.^158^ The discovery of MHC-related genes makes the definition of MHC unable to meet the traditional needs. So MHC is redefined as xMHC (the extended major histocompatibility complex). In addition to classical MHC-I, -II and -III, the flanking extended regions are also included. Duplication, polymorphism and linkage disequilibrium (LD) are the characteristics of xMHC. Paralogous copies are the important mechanism of diversity in the evolution of immune genes including xMHC genes.^159^ The significance of MHC paralogous group formation is to produce adaptive immune system.^160^ Human HLA class I and CLASS II are highly polymorphic, among which HLA-B is the most polymorphic gene. MHC polymorphism contains single nucleotide polymorphism (SNP) and deletion/insertion polymorphisms (DIPs).^159^ This polymorphism causes differences in MHC genes between different individuals, which is the key to the recognition of non-self components by immune cells of allogeneic organ transplants or grafts. Innate and adaptive immune cells have recognition receptors for MHC molecules. The interaction between the MHC antigen ligand of foreign allogeneic tissue cells and the specific receptor of host immune cells mediates the acute or chronic rejection of recipients to allogeneic organ transplantation. Acute rejection is of great significance for the early survival of grafts, while chronic rejection caused by the continuous effect of immune cells on ‘non-self’ antigens is an obstacle to the long-term survival of grafts. In addition, graft-versus-host disease caused by the immune response of the remaining immune cells of the graft (such as liver) to the recipient antigen is an important threat to the success of transplantation. The core is the formation of MHC polymorphism between different individuals. The negative selection of T cells makes them resistant to self- MHC components, while the positive selection makes them positively react to ‘non-self’ components, so as to protect the body from foreign antigens. But in the same organ transplantation, this protection becomes the key to affect the survival of the graft. Besides classical MHC, expanded MHC also plays an important role in transplantation immunity. In summary, xMHC and its interaction with immune cell receptors are of great significance for the activation of immune signals during transplantation.

## The receptors sensing non self

### TCRs

The allogeneic antigen peptide-MHC complexes carried by allograft organs provide the first signal for T lymphocyte activation in recipients. T cells recognize ‘non-self’ antigens in three ways: direct recognition, indirect recognition and semi-indirect recognition. Host T cells can directly recognize pMHC expressed on the donor APCs, and this recognition occurs rapidly without the presentation of the host’s own APCs, which is of great significance in the acute rejection at the early stage of transplantation. Autoantigenic peptides presented by the donor APCs or exogenous antigenic peptides (microbial origin), the exogenous peptide-MHC complex is cross-recognized by the recipient’s preexisting memory T cells (antigenic peptides from the same microbial origin). However, donor-derived APCs do not exist for a long time after transplantation, and host NK cells or cytotoxic T cells recognize the donor APCs as non-self-components, which are cleaved and cleared.^161,162^ Recipient-derived DCs then replace donor-derived DCs to play a sustained role after transplantation. Polymorphic proteins such as MHC are uptaken by host DCs and processed into heterologous antigen peptides. pMHC is formed with host MHC and expressed on APCs, which is recognized by recipient T cells. It is an indirect recognition pathway of allogeneic antigen by T cells. In addition to direct recognition and indirect recognition, T cells can recognize allogenic antigens through semi-indirect recognition. Donor DCs carries exosomes released by the graft to migrate to lymphoid tissue, and then exosomes are in vivo or attached to the host DCs cells for T cell recognition. DCs cells that captured donor exosomes expressed more endogenous MHC class II (IAb), CD40, CD80, and CD86.^163–165^

The recognition of pMHC by TCR alone is not sufficient to activate T cells. Costimulatory molecules are also necessary to provide the second signal to determine whether T cells are activated or inhibited. With the discovery of many families of costimulatory molecules, a deeper understanding of costimulatory molecules has been gained. CD28 is a costimulatory molecule expressed in CD4^+^T cells, and mainly in CD8^+^T cells. It interacts with B7 ligand through the recognition motif of extracellular MYPPPY.^166^ CTLA-4 (cytotoxic T lymphocyte antigen-4), which is structurally related to CD28, interacts with B7 molecules in the same way as CD28. However, CTLA-4 is induced after cell activation and transmits negative regulatory signals through various mechanisms to mediate T cell inhibition.^167^ TNF family members such as CD40-CD154 also play an important role in the proliferation and activation of T cells. Inhibition of CD154-CD40 interaction weakens the proliferation and differentiation of allogeneic CD4^+^ and CD8^+^ effect T cells. Antagonistic effect of CD154 on transforming CD4^+^ T cells into Foxp3^+^ peripheral Treg cells and increasing Treg cells accumulation in allografts.^168^ Various types of costimulatory molecules have been described in several reviews and will not be described here.^169,170^ In conclusion, the interaction between the stimulation and inhibition signals of costimulatory molecules determines the final state of T cells.

Cytokines secreted by APCs, such as IL-1, IL-6, and IL-12, function as the third signal of T cell activation, proliferation and differentiation, and IL-2 secreted by activated T cells regulates its biological effects by binding to corresponding cell surface receptors.^171^ T cells can differentiate into Th or CTL, and a small number of T cells can be transformed into memory T cells. CD8^+^ CTL cells induced apoptosis of target cells through degranulation (perforin, granase B) and death receptor (Fas/FasL).^172^ CD4^+^T cells differentiate into helper T cells under the influence of local microenvironment. Th1 cells induce delayed hypersensitivity (DTH) in allotransplantation, which is characterized by macrophage infiltration. Th1 cells secrete IFN-γ and TNF to stimulate macrophage activation and promote inflammatory response.^173^ Activated macrophages secrete IL-12 to further promote Th0 cells to differentiate into Th1 cells and form a positive feedback effect.^174^ Th2 secretes cytokines such as IL-4, IL-5, IL-10, and IL-13, which are commonly thought to inhibit cell-mediated immune responses and DTH responses. However, the study found that it is associated with antibody mediated rejection, and its secretion of IL-4 and IL-5 triggers eosinophil mediated rejection.^172^

### BCRs

B cells are activated primarily in a manner dependent on T cells. After allotransplantation, alloantigens are transported to secondary lymphoid organs and recognized by BCR as the first signal of immature B cells. These immature B cells can recognize both isoantigens expressed on the DCs membrane surface and soluble isoantigens. After being recognized and endocytosed by BCR, antigen peptides are formed through intracellular degradation pathways, and presented on the cell surface with MHC-II molecules. High affinity antibodies produced by B cells require interaction between T cells and MHC class II peptide complexes expressed on B cells.^175^ Except antigen recognition, the further activation of B cells also requires the participation of co-stimulatory signals. The crosstalk between Th cells and B cells provides multiple co-stimulatory signals for the activation of B cells. For example, CD40L on T cells interacts with CD40 expressed by B cells to induce B cell proliferation, antibody affinity maturation and Ig class switch. Cytokines secreted by Th cells provide the third signal for B cells, and the three signals participate in the proliferation and differentiation of B cells.^176^ Naїve B cells in the T-B border differentiate short-lived plasma cells and memory B cells and induce antibody production. T Follicular Helper Cells (Tfh) cells present in the germinal center (GC) are essential for the proliferation and differentiation of B cells. Tfh cells were identified with ICOS, CXCR5 and PD-1 as surface markers. Tfh cells expressing CXCR5 were induced to migrate to B cell follicles in a CXCL13-dependent manner, and BCL-6 transcription factors were induced by ICOS costimulatory molecules, thus promoting the differentiation of Tfh cells and effector cells.^177^ The cognate interaction between Tfh cells and B cells in GC promotes the proliferation and differentiation of long lived and short-lived plasma cells and memory B cells, as well as the production of antibodies. Cytokines such as IL-21, IL-4 and IFN-γ secreted by Tfh cells play an important role.^178^

Plasma cells, as terminal differentiated B cells producing antibodies, have a crucial role in antibody-mediated rejection. Plasma cells secret an antibody called IgM at first, but then isotype switching to IgG. IgG has a higher affinity, and plasma cells that produce high-affinity antibodies have a major advantage in rejection. Antibodies to allogenic MHC class I or Class II antigens, whose Fc segment interacts with complement or Fc receptor, exert immune damage to allografts through opsonization, classical complement activation pathway, and antibody-dependent cell-mediated cytotoxicity (ADCC).^179^

### Immunoglobulin-like receptors

TCR and BCR expressed by T and B lymphocytes are vital receptors that mediate the activation of adaptive immune system in transplantation. It is noteworthy that some immunoglobulin-like receptors present in immune cells also play a very important role in transplantation. Immunoglobulin-like receptor family is a kind of transmembrane receptor expressed in lymphocytes and myeloid cells characterized by extracellular C2 type Ig-like domains.^180^ As the first line of human immune defense, innate immune cells such as NK cells, macrophages and monocytes respond to exogenous pathogenic microbial antigens and endogenous danger signals, which is of great significance in the initiation and effect of inflammatory response. They also play an important role in allogeneic transplantation. Various types of immunoglobulin-like receptors are expressed on NK cells, monocytes, and macrophages, and they have outstanding contributions to the immune tolerance and immune response of antigen derived from allogeneic cells. NK cells are cell-killing innate immune cell types that express a series of activating and inhibiting receptors that regulate cell activation and function. NK cells express CD94/NKG2A, killer cell immunoglobulin like receptors (KIRs), LILRB1, and other human leukocyte antigen (HLA) class I specific inhibitory receptors, which play a crucial role in inducing self-tolerance in NK cells and their response to alloantigens.^181,182^ Activating and inhibitory LILRs expressed by myeloid cells are also involved in mediating graft tolerance and rejection. Studies have shown that some immunoglobulin-like receptors expressed by NK cells and myeloid cells, such as PIR-A and Ly49, actively participate in the specific memory of innate immune cells and may play a positive regulatory role in inducing chronic rejection.^183–185^ In addition, other receptors such as Siglec and signal regulatory protein α (SIRPα) expressed on immune cells are also important in the regulation of immune cell responses. Different from the above, the PSGs is a member of immunoglobulin superfamily produced and secreted by placental syncytial trophoblast cells to maternal blood during embryonic development, which is involved in inducing maternal and transplant immune tolerance. It may be a therapeutic target to induce transplantation tolerance

### Leukocyte immunoglobulin-like receptors

Leukocyte immunoglobulin-like receptors (LILRs) is located on human chromosome 19 and is widely expressed in lymphocytes and myeloid cells. LILRs contain five inhibitory receptors (LILRBs), five activated receptors (LILRAs) and one soluble (LILRA3) form.^186,187^ The expression of different types of LILRs regulates various biological processes.^188^ LILRBs and LILRAs are usually expressed on the cell surface in pairs. LILRAs (except LILRA3) are associated with FcRγ containing the immunoreceptor tyrosine-based activation motif (ITAM), and activate Syk/ZAP70 family kinases via ITAM to transmit positive signals.^189^ The tail of LILRBs contains an immunoreceptor tyrosine suppressor motif (ITIM), which recruits phosphatase SHP1/SHP2/SHIP in the Src homologous domain 2 to transmit negative signals.^190–192^ LILR plays an important role in inducing immune tolerance and immune response. For example, upregulation of LILRB2 and LILRB4 produces tolerant DCs, resulting in reduced expression of costimulatory molecules that induce T cell activation, resulting in T cell anergy.^193^ LILRB1 and LILRB2 has high affinity to HLA-G and their interaction is involved in inducing transplantation tolerance.^194^ In addition, LILRs are polymorphic among individuals, so it is reasonable to speculate that LILRs may cause immune rejection between the donor and recipient of allogeneic transplantation.^188^ This speculation has been verified in hematopoietic stem cell transplantation. The difference between the donor and recipient exerts specific LILRB3 antibody, which mediates graft-versus Leukemia (GVL)effect, however, specific LILRB3 antibody was not detected in renal allograft recipients.^195^

PIRs are human LILRs homologs located on mouse chromosome 7, including activating receptors (PIR-A) and inhibitory receptors (PIR-B). PIR-A and PIR-B is widely expressed in B cells, macrophages, mast cells, granulocytes and DCs.^196^ And they bind to MHC class I molecules to mediate the activation or inhibition of immune signals. PIR-B expressed on DCs inhibits the association of CD8 with MHC class I molecules and inhibits CTL triggering.^197^ PIR-B recruits SHP-1 through cytoplasmic ITIM tyrosine phosphorylation to negatively regulate B cell and myeloid cell activation.^198,199^ Unlike PIR-B, PIR-A induces positive regulation of immune signals via tyrosine phosphorylation to antagonize PIR-B and stimulate activation of myeloid cells.^200^ PIR-A indirectly participates in the initiation of T lymphocytes by promoting the activation of dendritic cells and inducing the proliferation of CTL cells. Besides, PIR-A is crucial in the production of specific innate immune memory of myeloid cells. Host myeloid cells (monocytes and macrophages) can obtain immune memory of non-self MHC class I antigens, which is manifested in the enhanced immune response when they are exposed to non-self MHC class I antigens again. This immune memory could be maintained for several weeks and is inhibited by the blocking of PIR-A. Deletion or blocking of PIR-A inhibits allograft rejection, suggesting that PIR-A may be a potential therapeutic target for allograft rejection.^185^

### Killer cell immunoglobulin like receptors

KIRs is a highly polymorphic gene family encoding activation or suppression receptors, expressed mainly in NK cells and also in T lymphocyte subsets (such as CD4, CD8 T cells and γδT cells^201^). Activating and inhibitory KIRs receptors transmit activation and inhibition signals through the immunoreceptor tyrosine activation motif (ITAMs)/immunoreceptor tyrosine based inhibitory motif (ITIM), respectively. KIRs (such as KIR2DL2/3) primarily bind to classical MHC class I molecules.^202,203^ Under physiological conditions, NK cells learn the normal expression level of MHC I through KIRs and Ly49 (homologous of KIRs in mice), and inhibit NK cells to kill cells with normal expression of MHC I by binding with MHC I to induce NK cells tolerance to healthy cells.^183,204^ Allogeneic graft expression, however, does not match with the host of HLA class I molecules, leaving the host NK cells KIRs lacking class I HLA inhibitory ligands, coupled with the transplant process factors such as virus and ischemia/reperfusion induced NK cell activated receptor and the ligand increases, causing the activation of NK cells and inhibitory signals imbalances, which manifested the killing effect on grafts.^203^ Ly49D is an activated receptor expressed on NK cells. The stimulation of Ly49D receptor by alloantigen leads to the proliferation and differentiation of NK cells. It should be noted that alloantigen stimulation alone is not sufficient to induce the expansion and differentiation of Ly49D^+^ NK cells. The inflammatory environment is also required to provide the necessary growth and differentiation factors. The expansion of Ly49D^+^ NK cells could be inhibited by the activated Ly49A receptor.^205^ Ly49A is an inhibitory receptor expressed on NK cells, and the transgenic expression of Ly49A inhibits the rejection of irradiated mice allogeneic bone marrow transplantation.^206^ However, a study shows that although depletion of Ly49A^+^NK cells partially affected the ability of B10, there was no significant change in the rejection of bone marrow cell allografts (BMC). But the depletion of both Ly49A^+^ and Ly49G2^+^NK cells significantly eliminated the rejection reaction. This shows the synergistic effect of Ly49A and Ly49G2 on the rejection of BMC.^207^ In addition, KIRs may play an important role in inducing specific immune memory NK cells. The use of immunosuppressants after organ transplantation increases the risk of viral infection, including cytomegalovirus (CMV). NK cells play the role of killing target cells of infected viruses in antiviral immunity and may produce memory NK cells for CMV-specific antigens. In a murine cytomegalovirus (MCMV) infected model, the activation receptor Ly49H interacts with m157 protein of MCMV to induce the production of memory NK cells, forming a memory pool containing a small number of memory NK cells. In the presence of semi-antigen or antigenic peptide presented by APCs, the Ly49C/I expressed by mouse NK cells binds to MHC class I molecules, inducing the production of liver-specific memory NK cells. The formation of this memory depends on the expression of CXCR6, which is another way to form memory NK cells.^183,184^

In addition to classical MHC I, KIRs can also bind to non-classical MHC I. Unlike other KIRs, KIR2DL4 is an evolutionarily conserved gene that recognizes Met^76^ and Gln^79^ in the α1 domain of HLA-G.^208^ HLA-G act as a ligand of KIR2DL4. Soluble HLA-G interacts with KIR2DL4 and is subsequently detected in endosomes, inducing activation of NK cells in different signal transduction modes and promoting secretion of cytokines and chemokines.^209^ But KIR2DL4 binds to HLA-G, a specific ligand derived from fetal trophoblast cells, induce transient tolerance in NK cells.^210^

KIRs are also expressed on CD8^+^T cells. Inhibitory KIRs could regulate TCR signal and inhibit CD8^+^T cell response, while activating KIRs could enhance functional T cell response. KIRs are only expressed in cloned and amplified terminally differentiated CD8^+^T cells, which are controlled by a single inhibitory or activated KIR. Different from NK cells, KIRs expressed in CD8^+^T cells is not subject to the functional education of HLA class I molecules, and KIRs expression down-regulates the functional response of CD8^+^T cells in an HLA-independent manner.^211^ For CD4^+^T cells, KIR2DL1 recognizes and binds HLA-CW7, mediating two opposite signals. Inhibition signals are generated by recruiting SHP-1 and SHP-2 under the synergistic effect of KIR2DL1 and TCR. In contrast, KIR2DL1, which only recruits SH-2, co-stimulates IL-2 production in CD4^+^T cells.^212^

### Natural killer group 2

Natural killer group 2 (NKG2) generally forms a heterodimer with CD94, and is a conserved C-type lectin like receptor in the immunoglobulin superfamily. NKG2 is expressed in NK cells and T cells subsets, including activating receptors and inhibitory receptors. As an inhibitory receptor, CD94/NKG2A recognizes class I HLA expressed in normal cells and is important in maintaining NK cells’ tolerance to normal cells, as well as killing abnormal cells with down-regulated HLA class I molecule.^213^ CD94/NKG2A could sense HLA polymorphism and transmit negative signals by ITIM.^182^ After transplantation, CMV infection induces differentiation of NKG2A^-^NKG2C^+^NK cells, which survive long and exert immune effect.^214,215^ CD94/NKG2D represents an activation counterpart of HLA Class I specific inhibitory receptors that transduces signals independently of ITAM and in a manner dependent on DAP10 adaptor molecules.^216^ The ligand MICA/B of CD94/NKG2D is induced to be expressed in the epithelium and endothelium of allografts during rejection.^217^ CD94/NKG2D ligand activates NK cells, provides T cell co-stimulation, and enhances antigen-specific CTL mediated cytotoxicity.^216^

### Sialic acid binding Ig like lectin

Sialic acid-binding Ig-like lectins (Siglec) are a class of negative immune regulators in the immunoglobulin (Ig) superfamily. Many Siglecs intracellular domains contain ITIM. Siglecs are expressed on most leukocytes with sialic acid containing glycans is used as ligand. Fourteen species have been identified in humans and nine in mice.^218^ Siglecs are involved in the regulation of innate and adaptive immunity in transplantation, autoimmune diseases, and tumors. The interaction between Siglec-10 on host APCs and CD24 on donor T cells inhibits the expansion and function of T cells, Reducing graft-versus-host disease (GVHD) in Allogeneic Hematopoietic cell transplantation.^219^ In addition to affecting T cell function, Siglecs can also induce tolerance of B cells. CD22 and Siglec-10 are two inhibitory receptors in the sialic acid binding immunoglobulin-like agglutinin (Siglec) family, whose binding to high affinity ligands induces B cell tolerance.^220^ Moreover, Siglec-10 regulates adaptive immunity by inhibiting the formation of MHC I-antigen peptide complexes cross-presented by DCs.^221^ The activation of NK cells was inhibited by siglec-7 binding to Sialylated glycans expressed on cancer cells.^222^ Siglec-15, as an emerging Siglec, could activate the cytoplasmic ITAM pathway and specifically bind to DAP12, thereby regulating the Syk signaling pathway.^223^ Siglec-15 was originally identified as involved in bone remodeling.^224^ The role of Siglec-15 in tumor immunity was subsequently discovered. Siglec-15 is upregulated in human cancer cells and tumor infiltrating macrophages/myeloid cells, participates in negative regulation of macrophage/myeloid cell immunity, and inhibits antigen-specific T cell responses.^225^ As such, Siglec-15 holds promise as a new approach for inducing transplantation tolerance.

### Signal regulatory protein α

SIRPα is an Ig superfamily receptor that is selectively expressed by myeloid cells. Its N-terminal V-set Ig domain interacts with the broadly expressed receptor CD47.^226^ The intracellular region of SIRPα has a typical immune receptor tyrosine suppressive motif (ITIM), which, upon phosphorylated, leads to recruitment and activation of SHP-1 and/or SHP-2, transmitting negative regulatory signals.^227^ SIRPα is considered to be a sensor capable of sensing the expression of CD47 in host cells, avoiding the activation of abnormal immune function by detecting self-signal. The interaction between SIRPα and CD47 can inhibit the function of macrophages and dendritic cells.^228^ SIRPα/CD47 axis plays an important role in the maintenance of transplantation tolerance. Myeloid derived suppressor cells (MDSC) are involved in the induction of transplantation tolerance (such as liver transplantation). Blocking either SIRPα or CD47 in MDSC reduces graft tolerance and induces graft dysfunction and rejection.^229^ The coding region of SIRPα gene is polymorphic, and the polymorphic of SIRPα variation mainly occurred in the V-like Ig domain interacting with CD47.^228^ The polymorphism of SIRPα expands the source of host recognition of ‘non-self’ antigens. The recognition of donor-derived ‘non-self’ signal SIRPα interacting with CD47 induces the activation of monocyte-positive signals in transplantation.^230^

### Pregnancy specific glycoproteins

Pregnancy-specific glycoproteins (PSGs) are members of the immunoglobulin superfamily and are located on chromosome 19q13.2. They are glycoproteins synthesized by placental syncytial trophoblast cells during pregnancy, generated during embryo implantation into the maternal blood and increase with time. They participate in immune regulation, thrombosis regulation and angiogenesis. Low levels of PSGs in the maternal circulation have been associated with spontaneous abortion, preeclampsia, intrauterine growth retardation, small for gestational age fetuses, and preterm delivery.^231–233^ PSG1 is the most widely studied member of PSGs, and has been determined to induce TGF-β activation.^234,235^ TGF-β inhibits cytotoxicity of CD8^+^T and natural killer cells and induces differentiation of CD4^+^Foxp3^+^Treg cells. Treg cells are critical for maternal immune tolerance to semi-allogeneic fetus and host immune tolerance to allografts.^236–238^ Recent studies have shown that TGF-β participates in the control of CD8^+^ Foxp3^-^Treg cell homeostasis which is significant in maintaining immune tolerance.^239^ In addition, PSG1 plays a role in preventing aGVHD. Recombinant PSG1 induces a significant increase in Foxp3 expression in naïve T cells in mice and humans, which is required to maintain the regulatory capacity of Treg. PSG1 treatment significantly inhibited aGVHD-related weight loss and mortality in a hematopoietic stem cell transplantation mice model.^240^ In addition to promoting Treg cell-associated immune tolerance, PSGs could induce monocytes and macrophages to secrete IL-10, IL-6, and TGF-β.^241,242^ These cytokines are important in inducing immune tolerance.

### Trained immunity: the maintenance of immune response during organ transplantation

**Fig. 4:**
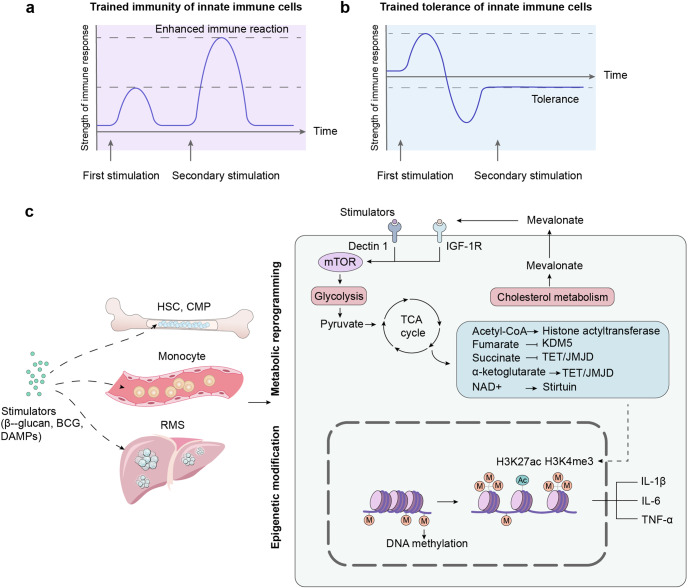
Trained immunity. **a**, **b** Exogenous or endogenous stimuli induce long-term functional reprogramming of innate immune cells, resulting in a stronger (**a**) or weaker (**b**) response to the second non-specific stimulation after the innate immune cells recover to the inactive state. **c** The inducer stimulates hematopoietic stem cells and myeloid progenitor cells (central) as well as blood monocytes and tissue macrophages (peripheral) to generate trained immunity. The reactive changes of inflammatory gene subsets in myeloid cells are mediated by metabolic and epigenetic reprogramming. Receptor signals from myeloid cells activate the AKT/mTOR/HIF-1α pathway, promoting the glycolysis. Its metabolite pyruvate enters the tricarboxylic acid cycle (TCA cycle), forming metabolic intermediates: Acetyl-CoA, Fumarate, Succinate, α-ketoglutarate, NAD^+^. These metabolic intermediates directly or indirectly mediate histone acetylation and methylation (H3K4me3, H3K4me1, H3K27ac)

Transplantation rejection is initiated by danger and stranger molecules sensing by their receptors. Recent advances highlight the critical role of the innate immune cells in chronic rejection. HMGB1 and Vimentin was reported to activate macrophages in unrecognized pathway associated with long-term activation.^243^ This long-term functional reprogramming of myeloid cells and macrophages has been termed ‘trained immunity’. As shown in Fig. 4, trained immunity refers to the capacity of innate immune cells to maintain their long-term function after PRR activation, which induces epigenetic, transcriptional, and metabolic programming. In lung, virus infection induced the trained immunity of alveolar macrophages on the mucosal surface, and promoted the long-term high expression of immune gene programs such as MHC-II, and the long-term high level of glucose metabolism. Even four months after virus infection, immune genes such as MHC-II were still at a high expression level.^244^ The live attenuated vaccine represented by BCG can also induce the trained immunity of monocytes and promoted monocytes to secrete more cytokines to the flavivirus vaccine.^245^ In addition, lipopolysaccharide (LPS), modified low-density lipoprotein (oxidized LDL or acetylated LDL), and even cytokines could also induce trained immunity of monocytes and macrophages.^246,247^ These studies have all confirmed that the ability of innate immune cells that response faster and stronger to specific or non-specific stimuli in a longer period of time.

**Fig. 5:**
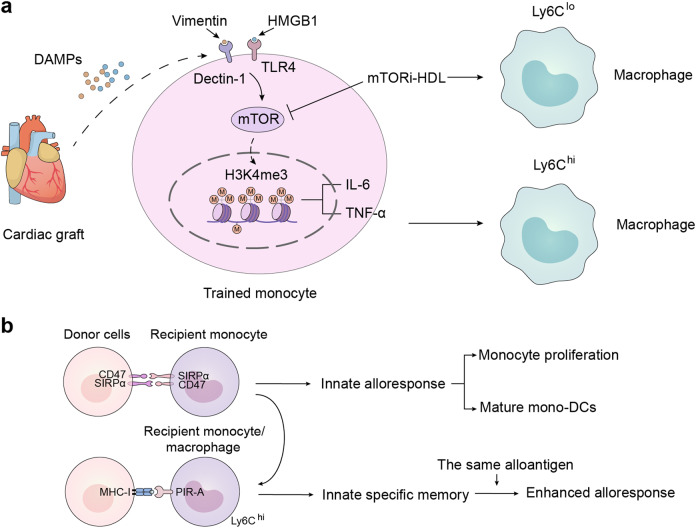
Innate immune memory in organ transplantation. **a** Ischemia and reperfusion events cause cell injury. Graft vascular endothelial cells, tissue cells, and some donor-derived immune cells are induced to release damage-associated molecular patterns (DAMPs) into the recipient circulation. Some cell molecules including Vimentin and HMGB1 interact with Dectin-1 and TLR4 receptors to reprogram circulating monocytes through the mTOR pathway (which may also include myeloid progenitor cells from bone marrow). The trained monocytes showed enhanced glycolysis and epigenetic modification. Trimethylation of the third lysine of histone at the gene encoding inflammatory factors mediates the transcriptional expression of inflammatory factors. These trained immune cells are characterized by high expression of Ly6C, and maintain the nonspecific memory of cells for a period of time. However, blocking mTOR by mTORi-HDL blockers can promote the transformation of monocytes into Mreg cells with low Ly6C expression. **b** Myeloid cells also exhibit specific memory characteristics of adaptive immunity. Donor-recipient polymorphism SIPRα is recognized by CD47 expressed by monocytes to produce an innate alloresponse. It is characterized by the proliferation of monocytes with high expression of Ly6C and the differentiation of mature mono-DCs. This interaction of SIPRα-CD47 may mediate myeloid-specific immune memory. Due to PIR-A is expressed in Ly6C^hi^ monocytes/macrophages. PIR-A recognizes polymorphic MHC-I antigens expressed by allogeneic transplanted cells and produces antigen-specific immune memory

Moreover, macrophages also exhibit a form of ‘memory’ immunity, in which macrophages, once immunized by allo-cells, undergo epigentic and transcriptional reprogramming, enabling them to directly recognize allograft and sustain the capacity to reject all-graft for a long term (Fig. 5). Our recent report identified PIRA as innate receptor to recognize allo-MHC I molecules.^185^ This term of memory or trained immunity of myeloid and macrophages may partly explain why the existing immunosuppressive schemes cannot successfully induce graft immune tolerance, nor prevent the chronic rejection.

As shown in Fig. 4b, hematopoietic stem cells, myeloid progenitor cells (central) as well as blood monocytes and tissue macrophages (peripheral) have the capacity to generate trained immunity.^248,249^ Upon stimulation, these myeloid cells undergo metabolic and epigenetic reprogramming. Its metabolite pyruvate enters the tricarboxylic acid cycle (TCA cycle), forming metabolic intermediates: Acetyl-CoA, Fumarate, Succinate, α-ketoglutarate, NAD+. These metabolic intermediates directly or indirectly mediate histone acetylation and methylation (H3K4me3, H3K4me1, H3K27ac).^250,251^

While most therapeutic approaches to prolong graft survival are targeting the adaptive immune cells, recent advances highlight the innate trained immunity. Blocking mTOR by mTORi-HDL blockers can prevent macrophage trained immunity and prolong graft survival.^252^ Targeting SIPRα-CD47 or PIR-A by blocking antibodies or fusion proteins also weaken the myeloid and macrophage memory capacity and prolonged allo-graft survival (Fig. 5).

### T cell memory and exhaustion: the balance of immune response to allo-graft and tumor cells

The immunotherapy has shown a powerful capacity to control the tumor using inhibitory molecule blockage antibody, such as anti-PD-1, anti-CTLA4 antibodies.^253^ But this brings challenge for transplant patient. Lipson et al.^254^ reported a case that anti-PD-1 antibody induced allograft loss, although it invigorated immunity against tumor in a kidney transplant patient. Moreover, Murakami et al. reported that 40% of 69 kidney transplant patients developed acute rejection after immune checkpoint blockades.^255^ Lots of single cell sequencing data show immune checkpoint blockades changed the subset of memory or exhausted T cells.

T cell memory and exhaustion is contrasting phenomena. Exhausted T (Tex) cells express high levels of inhibitory receptors such as PD-1, TIM-3, LAG-3, and TIGIT, produce less cytokine and loss the ability to control infections and tumor.^256^ Although these cells were well described in chronic infection and tumor, the Tex cells in transplant are less understood. Tex cells were observed in tolerant liver transplant patients. And the frequency of Tex cells correlated with improved transplant outcome.^257^ Memory T cells are more sensitive to antigen, function more rapidly, produce more cytokines and control the infections and tumor. Recent advances bring the concept of T cell stemness as a basic mechanism that keep T cell self renew and sustain T cell memory, which were described as TCF1^+^ precursor population.^258^

During transplantation, it is essential to identify therapeutic approaches to induce memory T cells specific for alloantigens exhaustion, but promote memory T cells against virus and tumor cells stemness. Though there are lots of clinical trials and FDA-approved targeted drugs to inhibit T cell response and T cell memory response, More approaches should be applied to prevent graft loss and prolong organ long term survival.

### Immune response in Xenotransplantation

Benefit by the development of immune anti-rejection drugs, organ transplantation has achieved a qualitative leap. However, the proportion between the number of patients waiting for transplantation and the number of donor donations is seriously out of balance each year, resulting in a large number of patients suffering from end-stage diseases dying while waiting for transplantation. Donor shortage is always a non-negligible obstacle in transplantation field. Xenotransplantation is considered as an important way to solve the shortage of donors. At present, pigs have been recognized as the best source of xenogeneic organs. Despite the post-transplant immune rejection, coagulation and biosafety barriers caused by pig-derived donors have been continuously improved under gene editing technology and donor pig breeding technology, the current laboratory has survived for pig-to-baboon xenotransplantation for up to 945 days.^4^ However, from laboratory and clinical research results, most of the results are still unsatisfactory. A recently reported case of a clinical cardiac xenotransplantation patient survived for 2 months and the cause of death is not yet clear,^7^ which means that the exploration of cardiac xenotransplantation still needs to be deepened.

#### Preformed natural antibody

**Fig. 6:**
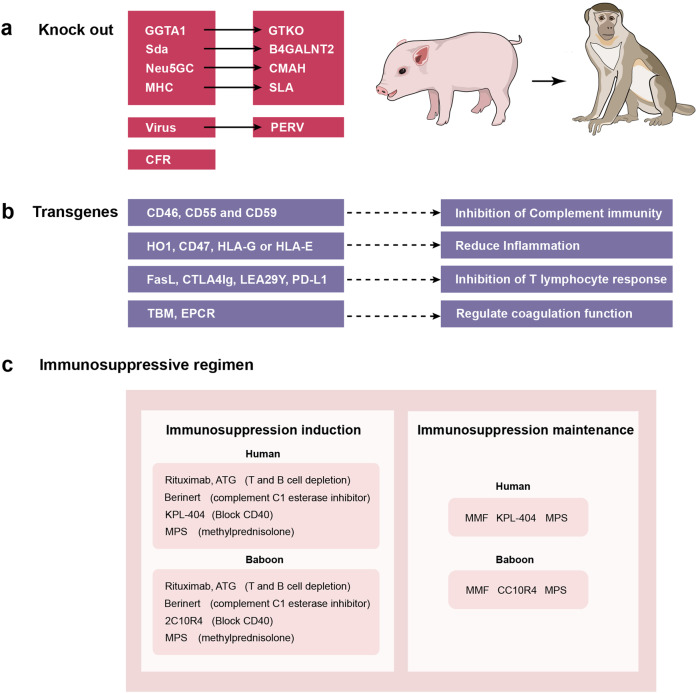
Xenotransplantation. **a** The knockout and transgenic pigs is used in xenotransplantation experiments that the pig organs are transplanted into the monkey. The knockout genes include GGTA1: α-1,3-galactosyltransferase, Sda: porcine blood type antigen, B4GALNT2: β-1,4-*N*-acetylgalactosamine transferase 2, encoding Sda antigen, Neu5GC: n-glycolylneuraminic acid, SLA: swine lymphocyte antigen, PERV: porcine endogenous retrovirus, CFR: growth hormone receptor. **b** The knockin genes include four group: inhibiting complement immunity: CD46, CD55, and CD59, reducing inflammation: HO1, CD47, and HLA, inhibiting T cell: FasL, CTLA4Ig, LEA29Y, and PD-L1, regulating coagulation: TBM(Thrombomodulin) and ERCP(Endothelial protein C receptor). **c** immunosuppresive regimen

Hyperacute rejection (HAR) occurs within minutes to hours after wild-type pig donor organs are transplanted into the human body without any human intervention. This is the result of rapid binding of porcine derived cell surface antigens to human preforming natural antibodies (NAbs) to trigger complement and coagulation cascade activation (Fig. 6). The most critical antigen is α-galactose-1,3-galactose (Gal), which is widely present in glycoproteins and glycolipids on the surface of porcine and most other animal cells. Gal is not synthesized in the human body because of a frameshift mutation in the human α-1,3-galactosyltransferase gene (GGTA1).^259^ Initially, Kozlowski et al. attempted to overcome heterogeneous HAR by removing natural anti-Gal antibodies. Although HAR did not occur in the recipient baboon, the level of natural antibodies returned to pre-perfusion levels in 4–6 days without kidney transplantation.^260^ It shows that the simple removal of NAbs does not prevent the production of new antibodies, which may lead to delayed graft rejection. With the birth of CRISPR/Cas9 gene editing technology, GGTA1 gene knockout pigs (GTKO) were successfully bred to effectively block Gal-mediated HAR from the root. In addition to Gal, there are a variety of cell surface antigens that bind to human Nabs on the surface of pig cells. It includes β-1,4 N-acetylgalactosaminyl transferase 2 (B4GalNT2), which can produce SDa erythrocyte antigen-like terminal carbohydrates, and a *N*-glycolylneuraminic acid (NeuGc) ligand expressed by glycoproteins and gangliosides. The pig B4GALNT2 gene has a conservative genomic structure and encodes an open reading frame with 76% amino acid homology with the human B4GALNT2 gene, widely expressed in porcine vascular endothelial cells. Cells expressing B4GALNT2 can induce human non-gal antibody response.^261^ NeuGc is highly expressed on the endothelial cells of all mammals except humans, and 85% of human serum contains natural antibodies against NeuGc.^262^ In order to prevent human preexisting NAbs from binding to these antigens, triple knockout pigs (TKO) have been successfully constructed: GGAT1, B4GalNT2, and CMAH (The cytidine monophosphate-n-acetylneuraminic acid hydroxylase). Deletion of porcine GGTA1/CMAH/β4GalNT2 gene indeed reduced the recognition of porcine heterologous antigens by human immunoglobulin.

#### Complement activation and coagulation dysfunction

Even though the binding of xenoantigens to human serum NAbs decreased after TKO, complement activation and coagulation pathway dysregulation were still observed after transplantation.^263^ Many factors can lead to complement activation and coagulation cascade initiation after xenotransplantation. The injury of pig donor endothelial cells caused by ischemia-reperfusion and the interaction between heterogeneous antigens and antibodies can promote complement activation and binding. This process can up-regulate the expression of adhesion molecules, promote platelet and leukocyte adhesion, and further aggravate vascular endothelial injury,^263^ leading to thrombosis. Normally, the human vascular endothelium expresses thrombomodulin (TBM) and endothelial protein C receptor (EPCR). TBM combined with thrombin can reduce the coagulation activity of thrombin and enhance its activity of activating protein C. Activated protein C (APC) has anticoagulant effect, making the blood state from procoagulant to anticoagulant.^264,265^ EPCR can specifically combine with protein C (PC) and activated protein C (APC), which improves the efficiency of PC activation.^266^ However, due to the heterogeneity between porcine endothelial-derived TBM/EPCR and human-derived proteins, the binding of porcine endothelial TBM and EPCR to human PC and APC cannot effectively regulate the excessive coagulation factor activation, which ultimately leads to thrombosis.^267^ In addition, CD39 expressed in pig endothelium can promote vasoconstriction and thrombosis by affecting adenosine metabolism. The abnormality of complement activation and coagulation function poses a great threat to the function and long-term survival of xenogeneic organs. Current strategies for inhibiting complement activation include reducing antigen-antibody binding and transferring human complement regulatory proteins such as hCD46, hCD55, and hCD59 that functions in the human circulatory environment. Human-derived CD46, CD55, and CD59 are expressed in porcine vascular endothelial cells, reducing the persistent activation of complement. Transgenic expression of hTBM, hEPCR, and hCD39 can inhibit the excessive activation of coagulation pathway and contribute to the long-term survival of xenografts (Fig. 6).

#### Adaptive response

The human immune system recognizes porcine xenoantigens similar to allogeneic transplantation but more responsive, especially indirect recognition.^268^ Direct T cell xenogeneic reaction is mainly targeted at SLA-DR molecules, which are preferentially recognized by CD4^+^T cells. The frequency of human anti-pig xenoreactive IL-2 secreting T cells was 10 times higher than that of anti-allogeneic stimulated cells.^269^ Kim et al. proved that CD4^+^ T cells play a more prominent role in xenotransplantation rejection in the pig-monkey kidney transplantation model. Selective clearance of CD4^+^ T cells rather than CD8^+^ T cells leads to long-term survival. In mixed lymphocyte reaction, the expression of SLA class II significantly increased the proliferation of CD4^+^ T cells, suggesting that SLA class II could be used as a donor gene modification target.^270^ Indirect recognition requires APC processing and presentation of the donor antigen on the recipient MHC molecule. Naive T cell populations are capable of generating strong indirect xenogeneic responses. And for a long time after the direct immunogenicity of the graft subsided, the recipient ‘s specialized APC still continued to process and present the graft antigen. Indirect rejection of T cells by nonhuman primates cannot be prevented by a strong immunosuppressive regimen of allograft rejection.^269^ This shows that T cell response has a stronger response to highly polymorphic pig antigens. Therefore, inducing T cell tolerance is a very important task. Currently, FasL,^271^ CTLA4Ig,^272^ LEA29Y (a human CTLA4-Ig derivate.),^273^ PD-L1^274^ and anti-CD2 monoclonal antibodies (Fig. 6)^275^ are transferred into donor pigs using transgenic technology to inhibit the co-stimulatory signal of T cells and enhance the inhibitory signal of T cells, so as to achieve the inhibition of T cells response in donor organs.

#### Innate response

As previously mentioned, innate immune cells such as NK cells and macrophages can recognize ‘self’ and ‘non-self’ antigens, usually resulting in tolerance to autoantigens and rejection to alloantigens. The molecular incompatibility of xenogenic and allogeneic antigens with recipient immune cells rejects the recognition of inhibitory receptors, leading to the rejection of donor cells by innate immune cells. Pig-derived donor cells have highly polymorphic xenoantigens, and are identified by innate immune cells such as NK cells and macrophages due to less similar surface markers, resulting in a stronger innate immune response.^276^ Macrophages and dendritic cells express SIRPα. CD47, as a ‘don’t eat me’ signal, interacts with the inhibitory receptor SIRPα and inhibits the phagocytosis of macrophages and dendritic cells.^277^ The cross-species molecular incompatibility of SIRPα-CD47 interaction leads to the rejection of xenogeneic target cells by macrophages. Human CD47 transgenic expression significantly reduces the phagocytosis of xenogeneic target cells mediated by human macrophages.^278^ Macrophages and NK cells express inhibitory receptors CD94/NKG2A that recognize MHC class I molecules. CD94/NKG2A recognizes HLA-E and HLA-G to inhibit cytotoxic effects.^279^ Molecular incompatibility between xenogeneic porcine MHC class I molecules and human immune cell receptors induces human cytotoxicity. Transgenic human HLA-E and HLA-G (Fig. 6) can significantly inhibit the cytotoxicity mediated by NK cells and macrophages,^280,281^ and inhibit the production of proinflammatory cytokines by macrophages.^282^

### Immunosuppressive therapy of xenotransplantation

The reported gene editing and immunosuppression regimens for xenotransplantation are not uniform, and the best gene editing and immunosuppression regimens have not been determined. Mohiuddin et al.described that xenotransplantation of cardiac allografts expressing human complement regulatory protein CD46 and human thrombomodulin α-1 – 3 galactosyltransferase gene knockout pigs (GTKO/hCD46/hTBM) survived more than 900 days in baboons. The immunosuppressive induction regimen for the recipient baboon included ATG (thymoglobulin), αCD20 antibody (rituximab), and αCD40 (clone 2C10R4). Cobra venom factor inhibits complement activation. The immune maintenance program includes MMF and αCD40 antibodies. When the earliest signs of rejection occur, methylprednisolone is injected intravenously for rescue treatment, and sufficient αCD40 antibody is given for rescue when rejection occurs. It is worth noting that this pig baboon xenotransplantation model is heterotopic heart transplantation.^4^ Recently, Mohiuddin et al.implemented the first 10-gene-edited pig xenograft heart transplantation in the human body. Gene editing includes three immunodominant heterologous antigen carbohydrate knockouts: GGTA1, B4GALNT2 and CMAH. To reduce intrinsic xenograft growth, growth hormone receptors are knocked out. To alleviate antibody-dependent complement damage and anticoagulant disorders, human CD46, CD55 and human TBM and EPCR were also expressed in pig donor cells. In order to reduce the inflammatory response, CD47 protein and heme oxygenase were expressed by transgenic. Immune induction regimen: rituximab, antithymocyte globulin, complement C1 esterase inhibitor (Berinert), humanized αCD40 monoclonal antibody (KPL-404) and methylprednisolone pulse therapy. Maintaining immunosuppression includes: mycophenolate mofetil, KPL-404, and rapid reduction of methylprednisolone (Fig. 6). The patient lost support after 7 weeks of cardiac xenograft. Porcine donors eventually developed unexplained sudden diastolic failure and generalized pathological myocardial thickening without systolic dysfunction. The mcfDNA test showed that pCMV was positive at a low level, but it could not be determined whether it would affect the life span of donor heart.^7^ It seems that the translation from laboratory to clinical, human and non-human primate species differences, porcine physiology and biosafety issues need to be explored in depth.

## Conclusions

Ischemia and reperfusion in organ transplantation causes cell metabolism reprogramming. Complex intracellular stress response promotes the production of multiple immune mediators. As mentioned above, hypoxia-sensitive factor HIF-1 is up-regulated in hypoxia and promotes the production of pro-inflammatory cytokines through various pathways. HIF-1 also regulates the transition of cell metabolism to anaerobic glycolysis, which results in lactic acid accumulation and leads to cell calcium overload mediated cell death with the participation of ion channels. The dead cells attract infiltration by immune cells and promote immune injury, partly due to the interaction between DAMPs and receptors. The metabolites of tricarboxylic acid cycle, ECT, and lipid metabolism caused by hypoxia/ischemia not only contain cytotoxicity, but also enhance immune response. In addition to metabolic changes, ER stress induced by hypoxia/ischemia plays an important role in regulating innate and adaptive immunity. For example, ER stress induced increased HSPs expression. When HSPs are released extracellular, they not only trigger inflammatory response, but also indirectly participate in adaptive response through dendritic cells. Graft reperfusion after ischemia, although necessary, exacerbates cell damage. Injured cells release a variety of DAMPs, such as HMGB1, ATP, HSPs, activates PRRs, induces inflammation cascade, and participates in the regulation of adaptive immune response. It is noteworthy, however, that DAMPs-induced DCs production supports T lymphocyte proliferation but does not drive T cell differentiation to dominate graft rejection.^2^

In the absence of immunosuppressants, the immune system recognizes ‘non-self’ alloantigens and strongly rejects the graft. Recognition of allogenic antigens by TCR and BCR plays an important role in the activation of T and B lymphocytes. T cells and B cells are differentiated into effector cells and memory cells, which have a continuous immune effect on the long-term exposure of allogeneic antigens in the host circulating immune system. Specific memory cells have a stronger rejection of pre-sensitive antigens. It was previously thought that this specific memory exists only in adaptive cells. Surprisingly, however, monocytes and macrophages can also form immune memory for non-self MHC-I antigens, and this memory response is closely related to the immunoglobulin-like receptor family member PIR-A. Furthermore, NK cells also produced specific memory responses to antigens through Ly49 receptors. The specific memory of innate immune cells for allogeneic antigens is of great significance for the chronic rejection of grafts. Immunoglobulin-like receptors (such as LILRs, KIRs, and NKG2) sense their own MHC molecules and protect healthy cells from immune cells, as well as distinguish allogenic antigens that mediate allogeneic rejection. Some ILRs genes are highly polymorphic. In allogeneic transplantation, the host develops an immune response to such allogeneic receptors, resulting rejection. However, some inhibitory immunoglobulins such as Siglec are involved in negative regulation of lymphocytes and myeloid cells. The interaction of SIRPα with CD47 can inhibit the function of macrophages and dendritic cells. In addition, PSGs produced during pregnancy are members of the immunoglobulin superfamily, mediating differentiation of Treg cells and inhibiting TCL. These inhibitory immunoglobulin-like receptors may play an important role in inducing transplant tolerance.
